# GCLiPP: global crosslinking and protein purification method for constructing high-resolution occupancy maps for RNA binding proteins

**DOI:** 10.1186/s13059-023-03125-2

**Published:** 2023-12-07

**Authors:** Wandi S. Zhu, Adam J. Litterman, Harshaan S. Sekhon, Robin Kageyama, Maya M. Arce, Kimberly E. Taylor, Wenxue Zhao, Lindsey A. Criswell, Noah Zaitlen, David J. Erle, K. Mark Ansel

**Affiliations:** 1 Department of Microbiology & Immunology and Sandler Asthma Basic Research Center, University of California San Francisco, San Francisco, CA USA; 2 University of California Berkeley, Berkeley, CA USA; 3 Department of Medicine, University of California San Francisco, San Francisco, USA; 4 Russell/Engleman Rheumatology Research Center, University of California San Francisco, San Francisco, USA; 5 Lung Biology Center, University of California San Francisco, San Francisco, USA; 6 School of Medicine, Sun Yat-Sen University, Guangzhou, 510080 People’s Republic of China

**Keywords:** Post-transcriptional regulation, RNA-binding proteins (RBP), T cells, Cis-regulatory elements

## Abstract

GCLiPP is a global RNA interactome capture method that detects RNA-binding protein (RBP) occupancy transcriptome-wide. GCLiPP maps RBP-occupied sites at a higher resolution than phase separation-based techniques. GCLiPP sequence tags correspond with known RBP binding sites and are enriched for sites detected by RBP-specific crosslinking immunoprecipitation (CLIP) for abundant cytosolic RBPs. Comparison of human Jurkat T cells and mouse primary T cells uncovers shared peaks of GCLiPP signal across homologous regions of human and mouse 3′ UTRs, including a conserved mRNA-destabilizing cis-regulatory element. GCLiPP signal overlapping with immune-related SNPs uncovers stabilizing cis-regulatory regions in CD5, STAT6, and IKZF1.

**Supplementary Information:**

The online version contains supplementary material available at 10.1186/s13059-023-03125-2.

## Background

The life cycle of protein-coding RNA transcripts involves their transcription from DNA, 5′ capping, splicing, 3′ polyadenylation, nuclear export, cellular localization, translation, and degradation [1–3]. RNA-binding proteins (RBPs) coordinately regulate these processes through interaction with RNA cis-regulatory elements, often in the 5′ and 3′ untranslated regions (UTRs) whose sequences are not constrained by a functional coding sequence [4]. Mammalian genomes encode hundreds of RBPs [5] and mutations in individual RBPs or even individual binding sites can induce strong developmental, autoimmune, and neurological defects in human patients and mouse models [6–9].

Post-transcriptional regulation plays an important role in T cell biology [10]. As much as half of the extensive gene expression changes that occur during T cell activation occur post-transcriptionally [11]. Over 1000 distinct RBPs have been identified in T cells [12] and several are known to be critical determinants of immune function and homeostasis [7]. A large proportion of probable causal genetic variants associated with immune-mediated diseases map to noncoding regions with potential regulatory functions in immune cells [13, 14], but the mechanistic role of the large majority of these variants in immune cells is unknown. A map of RBP occupancy in T cells can be a powerful tool for interrogating post-transcriptional gene regulation in the immune system and, in combination with genetic analysis, dissecting the genetic basis of immune-mediated diseases.

Systematic analyses of protein-RNA interactions have expanded our understanding of post-transcriptional regulatory circuits [5, 12, 15–22]. Large-scale enhanced crosslinking immunoprecipitation (eCLIP) studies provided invaluable information about RNA elements bound by > 150 specific RBPs in an accessible public database, the Encyclopedia of DNA elements (ENCODE) RNA-binding protein resource [19]. However, a much larger number of RBPs remain to be analyzed, and protein-specific assays are an inefficient means to interrogate global RBP occupancy across cell types and conditions. Methods utilizing organic phase separation to separate ribonucleoprotein complexes expanded the repertoire of known RBPs [5, 12, 15–17]. These and other RNA interactome capture studies [18, 20–22] have mostly focused on the *trans* factors involved in RNA regulation, but also provide information about ribonucleoprotein-associated RNA regions [19–22].

Here, we created global RBP occupancy maps for primary mouse T cells and the human Jurkat T cell line using Global Cross-Linking Protein Purification (GCLiPP). The GCLiPP method shares many technical features with eCLIP and produces the same high-resolution transcriptome-wide protein occupancy data without RBP-specific immunoprecipitation. We validated GCLiPP, benchmarked its performance, and demonstrated its utility for discovering and interrogating post-transcriptional cis-regulatory elements that impact gene expression and the incidence of human immune-mediated diseases. We present GCLiPP and the RBP occupancy maps it generates as resources for functional analysis of post-transcriptional regulation.

## Results

### Transcriptome-wide analysis of RBP occupancy in T cells

**Fig. 1:**
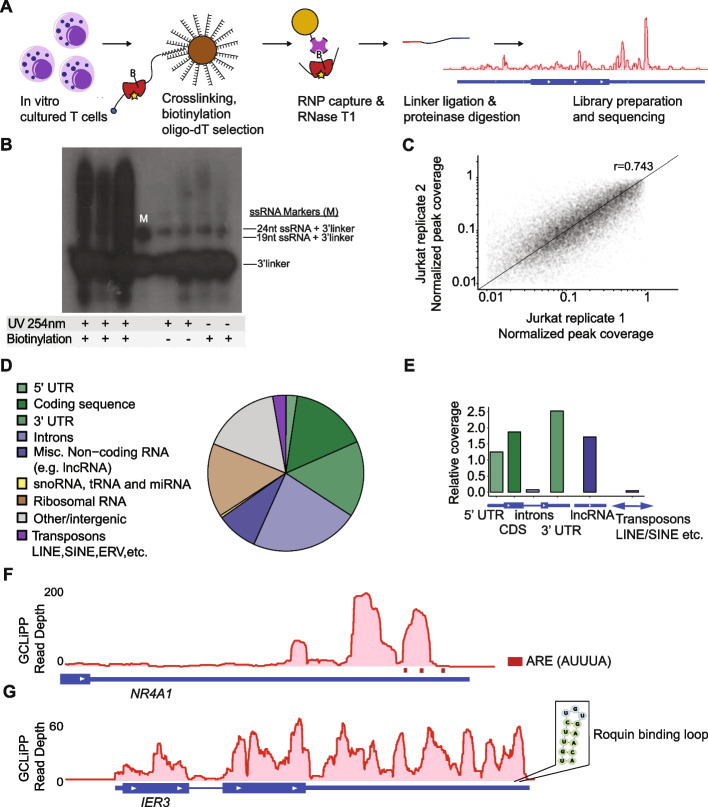
GCLiPP sequencing reveals RNA transcript protein occupancy. **A** GCLiPP method of global RBP profiling. T cell RNAs are crosslinked to RBPs and lysates are biotinylated on primary amines. mRNAs are enriched with oligo-dT beads, and RBP-protected sites are digested, captured, sequenced, and aligned to the genome. **B** Film image of RBP-bound RNAs captured from Jurkats that underwent either UV crosslinking (UV 254 nm), protein biotinylation, or both. Lane marked “M” contains 19 and 24 nucleotide (nt) ssRNA ligated to radiolabeled 3′linker. RNA greater than 24nt + 3′linker size were extracted and processed for sequencing. **C** Normalized GCLiPP read depth (fraction of reads in called peak relative to all GCLiPP reads in annotated 3′ UTR) in two replicates of Jurkat cells. ρ represents Pearson correlation. **D** Proportion of mapped GCLiPP reads derived from genomic features. **E** Relative coverage of genomic features in GCLiPP sequencing reads relative to total length of genomic features of indicated class. **F** GCLiPP track of *NR4A1* 3′UTR. Red bars indicate presence of ARE motif (AUUUA). **G** GCLiPP track of *IER3* gene along with predicted ROQUIN binding loop in the 3′UTR

To achieve transcriptome-wide RBP binding site profiling in T cells, we adapted biochemical methods for crosslinking purification of all mRNA-RBP complexes. Our Global CrossLinking Protein Purification method, abbreviated as GCLiPP, features crosslinking of endogenous ribonucleoprotein complexes using high-energy UV light (no photo-crosslinkable ribonucleotide analogs); oligo-dT pulldown prior to biotinylation to enrich for mRNA species; chemical biotinylation of primary amines using a water-soluble reagent with a long, flexible linker; brief RNase digestion with RNase T1; and on-bead linker ligation with radiolabeled 3′ linker to facilitate downstream detection of ligated products (Fig. 1A). We used the guanine-specific ribonuclease T1 to favor larger average fragment sizes than would be produced with an RNA endonuclease with less stringent nucleotide specificity, such as RNase A. We first applied GCLiPP to interrogate RBP-occupied regions of RNA in human Jurkat T cells. Linker-ligated RBP-protected fragments were separated by PAGE and detected by radiography (Fig. 1B, lanes 1–3). Single-stranded RNA oligonucleotides of 19 and 24 nt, the same length as the 5′ and 3′ linkers, were ligated to the radiolabeled 3′-linker and served as size markers (Fig. 1B, lane 4). Material greater than 24 nt + 3′-linker in length were predicted to contain RBP-bound RNA fragments, and these were extracted and processed for small RNA library preparation and sequencing. Excluding the protein biotinylation or UV crosslinking steps greatly diminished the yield of ligated RNA fragments (Fig. 1B, lanes 5–8), indicating that the GCLiPP procedure preferentially captures RNA sequences interacting with RBPs in living cells.

We called local peaks of GCLiPP sequence read density and measured the distribution of GCLiPP reads within those peaks to assess the reproducibility of the technique. Local read density within individual transcripts was similar between experiments, as GCLiPP fragments yielded highly reproducible patterns in technical replicates (Fig. 1C). The distribution of read coverage from Jurkat GCLiPP libraries was strongly enriched within mature mRNAs and long noncoding RNAs (Fig. 1D, E) compared to other transcriptome features.

RBPs bind to linear and structural motifs to regulate the stability and/or translation of the mRNAs that they bind [23]. We observed GCLiPP read coverage corresponding to known RBP recognition motifs. Nuclear Receptor subfamily 4 group A member 1 (*NR4A1*), which encodes the NUR77 protein that is an activation-induced negative regulator of T cell responses, is an example of RBP-mRNA interaction through linear sequence recognition. A local maximum of GCLiPP read density in the *NR4A1* 3′UTR corresponded with a region that contains multiple AU-rich elements (AREs) that destabilize mRNA (Fig. 1F) [24]. Similarly, the 3′UTR of *IER3*, an immediate early response gene that protects cells from Fas- or TNFα-induced apoptosis, contains a local maximum of GCLiPP read coverage at the previously characterized structurally determined stem-loop binding motif regulated by the RBP Roquin (Fig. 1G) [25]. These examples provide snapshots of different motifs represented in GCLiPP protein occupancy maps. Further examination of individual 3′UTRs of interest can be accessed through our visualization tool, Thagomizer (http://thagomizer.ucsf.edu). Thagomizer utilizes a database of GCLiPP and Argonaute 2 (Ago2) HITS-CLIP experiments [26, 27] along with miRNA binding site predictions from the TargetScan database [28] to map RBP-mRNA and miRNA-mRNA interactions in 3′ UTRs.

**Fig. 2:**
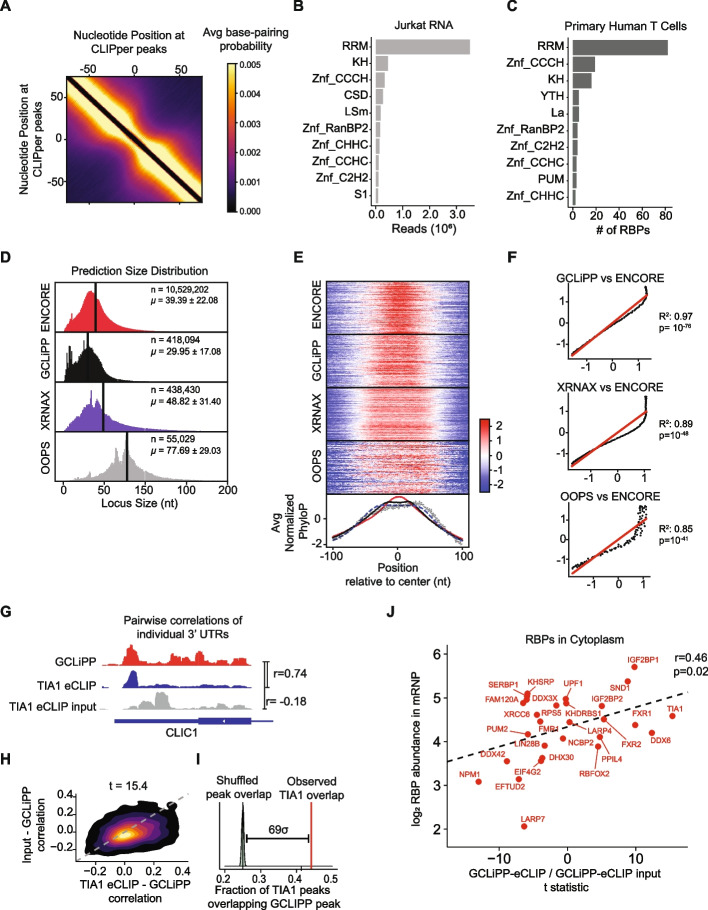
GCLiPP detects cytosolic RBP binding sites with characteristic sequence conservation and structural properties. **A** Base-pairing probability was calculated for each pair of nucleotides within 200 bp peak called by CLIPper2.0 in Jurkat cells. The average base-pairing matrices for all peaks in the 3**′**UTR is shown here as a heatmap. **B** Jurkat RNAseq reads mapped to known RBPs were categorized into different RBDs. Top 10 occurring domains were determined by total reads that can be ascribed to specific domain motif. **C** Number of RBPs identified through RNA-IC in activated primary human T cells that contain a certain domain. Only top 10 occurring motifs are shown. RBPDB databased was used as a reference for categorizing RBPs in **B** and **C**. **D** Size distribution of n CLIPper-called peaks from datasets of RBP binding detected by GCLiPP, phase-separation methods XRNAX and OOPS, and an amalgamation of 87 RBP eCLIP datasets from ENCORE. µ = mean ± standard deviation. **E** Sequence conservation of called peaks from **D** expressed as normalized PhyloP score relative to the peak center. Histograms at bottom show the global average for all peaks for each method. **F** Normalized PhyloP data from **E** transformed to display the correlation between eCLIP (y-coordinate) and the indicated methods (x-coordinate) as a function of the distance from peak center. Linear regression statistics and the line of unity (in red) are indicated on each plot. **G** Genomic snapshots of individual 3**′** UTR showing exemplary correlation between TIA1 eCLIP dataset and GCLiPP. GCLiPP is shown in red, while the indicated RBP eCLIP data is shown in blue, and matched control input samples are shown in gray for the 3**′** UTRs of the indicated gene. *r* indicates Pearson correlation between pairs of normalized read density at a given nucleotide for the indicated comparisons. **H** 2D density plots showing matched correlations between GCLiPP and TIA1 eCLIP (*X*-axis) and GCLiPP and the matched control input sample (*Y*-axis) for individual 3**′** UTR for all expressed genes in eCLIP and GCLiPP datasets. The *t*-statistic shown is for a paired *t*-test of the correlations. **I** Overlap of CLIPper-called peaks in 3**′** UTRs in GCLiPP and eCLIP. Red lines indicate observed overlap of GCLiPP peaks and eCLIP peaks. Gray distribution represents bootstrapped expected overlap, derived by computing overlap of GCLiPP-called peaks with eCLIP peaks shuffled within the same 3**′**UTR. This analysis was repeated 500 times. The indicated distance represents the number of standard deviations above the mean shuffled overlap of the observed overlap. **J** Correlation of eCLIP-GCLiPP paired *t*-tests from **H** and cytosolic RBP abundance in mRNPs

Systematic analysis determined that single-stranded RNA (ssRNA) was the dominant structural characteristic of protein-occupied RNA regions detected by GCLiPP. We used CLIPper [29] to call peaks in our data and calculated the base-pairing probability for every nucleotide pair in each 200-bp sequence peak using RNAfold in the ViennaRNA package [30]. Matrices for all peaks were averaged to generate an average base-pairing probability. This analysis revealed a decreased probability of base-pairing at the center of GCLiPP peaks compared to surrounding regions, indicating an enrichment for single-stranded RNA (ssRNA) at the center of GCLiPP peaks in Jurkat cell 3′UTRs (Fig. 2A). A similar pattern of decreased probability of base-pairing was observed in eCLIP peaks for a characteristic member of this family, Polypyrimidine Tract Binding Protein 1 (PTBP1), an RBP that binds to C/U-rich ssRNA through 4 RRM domains (Additional file 1: Fig. S1A) [31]. UV crosslinking bias may drive ssRNA capture; however, this enrichment in GCLiPP peaks was consistent with high expression of RBPs with ssRNA-binding RNA recognition motif (RRM) domains in Jurkat cells (Fig. 2B). Proteomics data from similar RNA interactome capture (RNA-IC) method in primary human CD4 T cells [12] also captured RBPs that predominantly contained the RRM motif compared to other domains (Fig. 2C). Together, these data indicate that RBP-occupied regions detected by GCLiPP in T cells are predominantly composed of the structural motif, ssRNA.

### GCLiPP read density represents cytosolic RBP occupancy

We assessed the performance of GCLiPP by comparison with eCLIP and phase separation-based methods. Specifically, we compared CLIPper-called peaks in Jurkat GCLiPP data with compiled peaks from eCLIP datasets [32], and with peaks detected in the original exemplary XRNAX [15] and OOPs [16]. CLIPper returned peaks of differing size distributions for each method (Fig. 2D; *p* < 10^−300^ for all pairwise comparisons). Phase separation methods, especially OOPS, generated broader peaks, possibly indicating lower-resolution mapping of RBP-occupied regions. To better assess assay resolution, we determined the phylogenetic conservation of RBP-occupied regions detected by each technique, reasoning that functional RBP-RNA interaction sites are better conserved than neutral 3′ UTR sequences. PhyloP scores for 200 nt regions centered on each CLIPper-called peak were averaged for all binding sites and then normalized around a mean of 0 for each method (Fig. 2E). GCLiPP and eCLIP peaks displayed high sequence conservation at peak centers, although GCLiPP showed a slightly broader local maximum of conservation. XRNAX and OOPS produced even broader patterns of phylogenetic conservation, indicating lower-resolution mapping of RBP binding sites, consistent with the broader distribution of sequence reads generated by these methods. Normalized PhyloP scores at each nucleotide distance from peak center correlated better between eCLIP and GCLiPP (Fig. 2F, top panel) than between eCLIP and phase separation methods (Fig. 2F, middle and bottom panel). We conclude that GCLiPP globally and selectively detects RBP binding sites throughout the transcriptome at a high resolution that closely resembles gold-standard eCLIP data.

Given the global similarity between eCLIP and GCLiPP, we systematically compared GCLiPP occupancy maps with individual eCLIP experiments [32]. We examined pairwise correlations of normalized read density across individual 3′ UTRs between GCLiPP and individual RBP eCLIP samples (Fig. 2G, Additional file 1: Fig. S1B). In parallel, we compared GCLiPP to the input control for each eCLIP experiment. Since the eCLIP input controls ideally report all crosslinked ribonucleoprotein complexes, albeit with low coverage and a low signal to noise ratio, we expected GCLiPP to broadly correlate with the input. Nevertheless, eCLIP for many RBPs, such as TIA1 and IGF2BP1, matched GCLiPP read density much more closely than the eCLIP input control across the transcriptome (Fig. 2H, Additional file 1: Fig. S1C), indicating a relatively high contribution of these RBPs to the overall GCLiPP signal. For other proteins, such as PUM2, this comparison showed poor correlation, indicating a low contribution to total RBP occupancy transcriptome-wide. Yet we found evidence that GCLiPP captured focal RBP binding to specific sites (UGUA motifs in the case of PUM2) that were overrepresented in GCLiPP reads (Additional file 1: Fig. S1B, bottom panel). This was revealed when we called GCLiPP peaks with CLIPper [29] and compared these peaks with CLIPper-called peaks in eCLIP datasets. The observed fraction of PUM2 eCLIP peaks that overlap GCLiPP peaks (0.56) was much greater than the fraction overlapping eCLIP peaks randomly shuffled across the 3′ UTRs from which they were derived (Additional file 1: Fig. S1D, bottom panel). Similar results were obtained for TIA-1 (Fig. 2I) and IGF2BP1 (Additional file 1: Fig. S1D, top panel). These enrichments above background binding for IGF2BP1, TIA1, and PUM2 were among the highest 8 of the 87 RBPs whose eCLIP signals were examined (Additional file 1: Fig. S2).

These analyses indicated that GCLiPP captures RNA occupied by any protein. If so, the most abundant RBPs should generally make greater contributions to the GCLiPP signal than less abundant RBPs. Therefore, we further compared the genome-wide correlation between eCLIP and GCLiPP signal with the abundance of these 87 RBPs as previously determined via mass spectrometry [21]. There was an overall significant correlation between RBP abundance and correspondence between RBP eCLIP and GCLiPP profiles (r = 0.28, *p* = 0.02). However, stratifying RBPs by their predominant cellular localization [33] showed that this correlation was driven almost entirely by cytosolic RBPs with no correlation for non-cytoplasmic RBPs (Fig. 2J, Additional file 1: Fig. S1E). The fraction of eCLIP peaks that overlapped GCLiPP peaks above a shuffled background was also significantly greater for cytosolic versus non-cytosolic RBPs (*p* = 0.003, Additional file 1: Fig. S2 inset). These findings were expected, as the GCLiPP experimental protocol preferentially samples the cytosol by eliminating most nuclear material in the cell lysis step. In summary, GCLiPP and eCLIP represent similar and complementary methods for high-resolution mapping of RBP occupancy on cytosolic RNAs.

### Comparison of RBP binding profiles of different T cell states

**Fig. 3:**
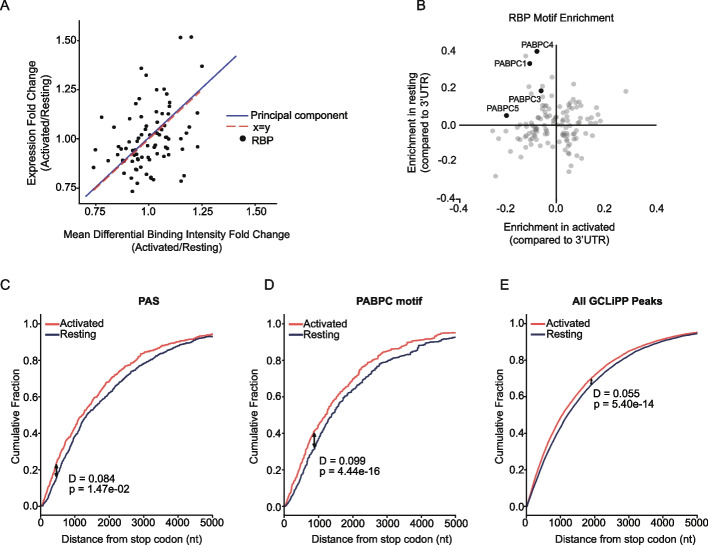
Activation-induced changes in RBP binding. **A** Correlation between activation-induced changes in RBP expression and protein occupancy at corresponding binding sites in Jurkat cells. Mean differential binding intensities were calculated for sets of GCLiPP peaks defined by their overlap with specific RBP binding in ENCODE eCLIP datasets. Dots represent individual RBPs, and lines show the concordance between the first principal component (blue) describing variation within these data with the line of unity (red). **B** Enrichment of RBP consensus binding motifs centered within regions with increased GCLiPP signal in activated vs. resting Jurkat cells. Genomically encoded motifs recognized by 4 PABPC family members were enriched in regions differentially bound in resting, but not activated Jurkat cells. **C–E** Cumulative distribution function (CDF) plots depicting the distance from the translation stop codon for the top 10% of differentially bound regions in activated compared to resting Jurkat cells filtered for those containing a canonical PAS (**C**), genomic templated PABPC binding site (**D**), or any GCLiPP peak (**E**)

We further demonstrated the utility of GCLiPP through a series of experiments in T cells. Changes in RBP occupancy at any given genomic location can be affected by a variety of factors, including RBP expression and site availability. To compare RBP occupancy between different samples, we developed a deep-learning algorithm, DeepRNAreg, to identify regions of differential GCLiPP read density within each 3′UTR and applied it to data from unstimulated and stimulated Jurkat cells. DeepRNAreg calculates the area under the curve of the read coverage and assigns a differential binding intensity (DBI) value to the genomic location. Using DeepRNAreg, we identified differentially bound sites between resting and activated Jurkats (Additional file 2: Table S1-S2), then queried ENCORE eCLIP data to determine which RBP(s) bind to these genomic locations. Changes in binding intensity between activated and resting Jurkats mirrored changes in RBP expression (Fig. 3A), with higher DBI at sites bound by an RBP associated with higher expression of that RBP in activated vs resting cells. These data indicate that RBP expression is often a limiting factor for occupancy on transcripts, as higher expression is associated with greater occupancy across the binding site repertoire.

To determine whether any specific RBP-RNA interactions were enriched in either resting or stimulated conditions, we identified predicted RBP motifs within each differentially bound region using the oRNAment database [34], and determined the enrichment of the motif in either dataset compared to its normal occurrence within 3′UTRs of the human genome. Among the proteins examined, poly-A-binding protein cytoplasmic family (PABPC) motifs were enriched in resting, but not in activated Jurkat cells (Fig. 3B). However, PABPC proteins were not differentially expressed in these conditions, indicating that changes in binding site availability rather than protein abundance may drive this enrichment. PABPC proteins bind to the untemplated poly-A tail of transcripts, as well as to adenosine-rich motifs that are preferentially located near the 3′ end of 3′UTRs [35]. Activated T cells preferentially express shortened transcripts through utilization of upstream alternative polyadenylation signal sequences (PAS) (Fig. 3C) [35]. Therefore, we hypothesized that the reduced global binding to PABPC motifs may reflect a reduction in their availability in expressed transcripts. Indeed, the set of PABPC binding motifs differentially bound in resting Jurkat cells was significantly skewed toward those more distant from the translation termination codon (Fig. 3D). A similar but less pronounced phenomenon was apparent for all GCLiPP peaks (Fig. 3E). Together, these data indicate that global RBP occupancy in Jurkat T cells may be altered by activation-induced changes in RBP expression and PAS selection.

### RBP occupancy of RNA cis-regulatory elements in primary T cells

**Fig. 4:**
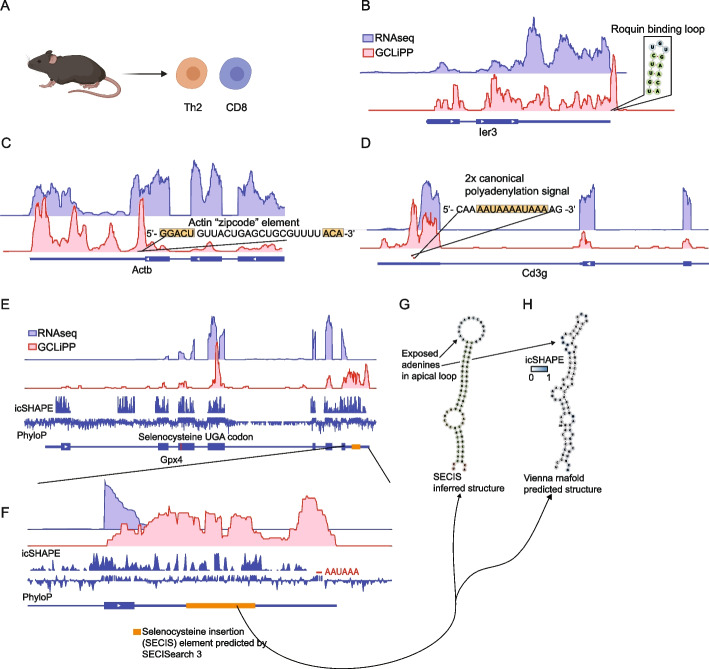
GCLiPP recapitulates previously described mRNA-RBP interactions in primary T cells. **A** GCLiPP was performed on primary mouse Th2 and CD8 T cells. RNAseq and GCLiPP tracks for **B** *Ier3*, **C** *Actb*, **D** *Cd3g*, and **E–G** *Gpx4*. RNAseq tracks are from resting Th2 cells. GCLiPP tracks show the sum of five experiments, three in Th2 and two in CD8 T cells. Location of known RBP binding determinants are shown as insets

Previous global RBP profiling has been conducted with cell lines. To examine transcriptome-wide RBP occupancy in primary T cells, we performed GCLiPP on primary mouse CD8 and CD4 type 2 helper T cells (Th2) (Fig. 4A). These two subsets of T cells perform different functions with CD8 T cells involved in cell-mediated immunity and Th2 cells involved in orchestrating barrier immunity. Despite these differences, the cells share core T cell machinery and were treated as a broader group of primary mouse T cells for the following analyses. Local read density at peaks showed reproducible patterns between multiple pooled experiments for the two T cell subsets (Additional file 1: Fig. S3A). Similar to Jurkat cells (Fig. 1D, E), distribution of reads in primary mouse T cells was enriched in mature transcripts and long noncoding RNAs (Additional file 1: Fig. S3B, C). The most striking difference was the greater proportion of reads derived from transposable elements in mouse GCLiPP libraries. This increase is likely due to the greater amount of annotated transposable elements in the mouse genome since the relative coverage of these elements was similar between species. We examined the GCLiPP profiles at previously characterized cis-regulatory elements of various functional and structural categories in primary mouse T cells. As in Jurkat cells, we observed GCLiPP read density at Roquin/Regnase binding site in the 3′ UTR of *Ier3* (Fig. 4B).

Known cis-regulatory elements involved in transcript localization were also represented by local regions of GCLiPP read density. The Beta-actin “zipcode” element is responsible for localization of *Actb* mRNA to the cellular leading edge in chicken embryo fibroblasts [36] and contains conserved linear sequence elements separated by a variable linker. These conserved sequence elements are thought to form the RNA/protein contacts in a complex involving the actin mRNA- and the RNA-binding protein Igf2bp1 (previously known as Zbp1) where the non-conserved sequence winds around the RBP [37]. This sequence corresponds to the center of the second highest peak of GCLiPP read density in the *Actb* transcript (Fig. 4C).

The canonical PAS (AAUAAA) binds to RBPs in the polyadenylation complex as part of constitutive mRNA metabolism [38]. We examined T cell lineage-defining transcripts with well-resolved GCLiPP profiles (due to their high expression levels), including *Cd3g* (Fig. 4D), *Cd3e*, *Cd4*, and *Cd8b1* (Additional file 1: Fig. S4). The canonical PAS in these transcripts were contained within called GCLiPP peaks, often as the peak with the highest GCLiPP read density in the entire transcript. Interestingly, the GCLiPP profile of *Cd8b1* contained direct biochemical evidence for alternative polyadenylation signal usage (Additional file 1: Fig. S4C), a phenomenon that has previously been described to be important in activated T cells [35]. GCLiPP peaks appeared in multiple canonical polyadenylation signal sequences in *Cd8b1*, coincident with clear evidence for both short and long 3′ UTR isoform usage indicated by lower RNAseq read counts after the initial canonical polyadenylation signal. A similar pattern was apparent in *Hifa* (Additional file 1: Fig. S4D) and a number of other highly expressed transcripts.

The insertion of the selenium containing amino acid selenocysteine into selenoproteins represents a unique case of RBP regulation of protein translation. Selenoproteins are redox enzymes that use selenocysteine at key reactive residues [39, 40]. Selenocysteine is encoded by the stop codon UGA. This recoding occurs only in mRNAs that contain 3′ UTR *cis*-regulatory elements (termed SECIS elements) that bind to RBPs that recruit the elongation factor Eefsec and selenocysteine-tRNA [41, 42]. SECIS elements were prominent peaks of GCLiPP read coverage in selenoprotein mRNAs. For example, the predicted SECIS element [43] in the 3′ UTR of *Gpx4* was entirely covered by GCLiPP reads (Fig. 4E). Indeed, a canonical polyadenylation signal and the full hairpin structure containing the SECIS element account for essentially all of the GCLiPP reads in the *Gpx4* 3′ UTR (Fig. 4F). Comparing transcriptome-wide in vivo folding data from icSHAPE [44] and GCLiPP data supports the identification of an RBP-bound, structured SECIS element (Fig. 4G,H). Furthermore, this analysis suggests that the folded, RBP-bound structure is even larger than that predicted by SECISearch 3, with regions of GCLiPP read density and apposed high and low icSHAPE signals spanning almost the entire 3′ UTR. Thus, GCLiPP recapitulated previously described structured and single-stranded RNA cis-regulatory elements that mediate constitutive RNA metabolism, transcript localization, regulation of gene expression, and translation.

### Cross-species comparison of GCLiPP reveals patterns of biochemically shared post-transcriptional regulation

**Fig. 5:**
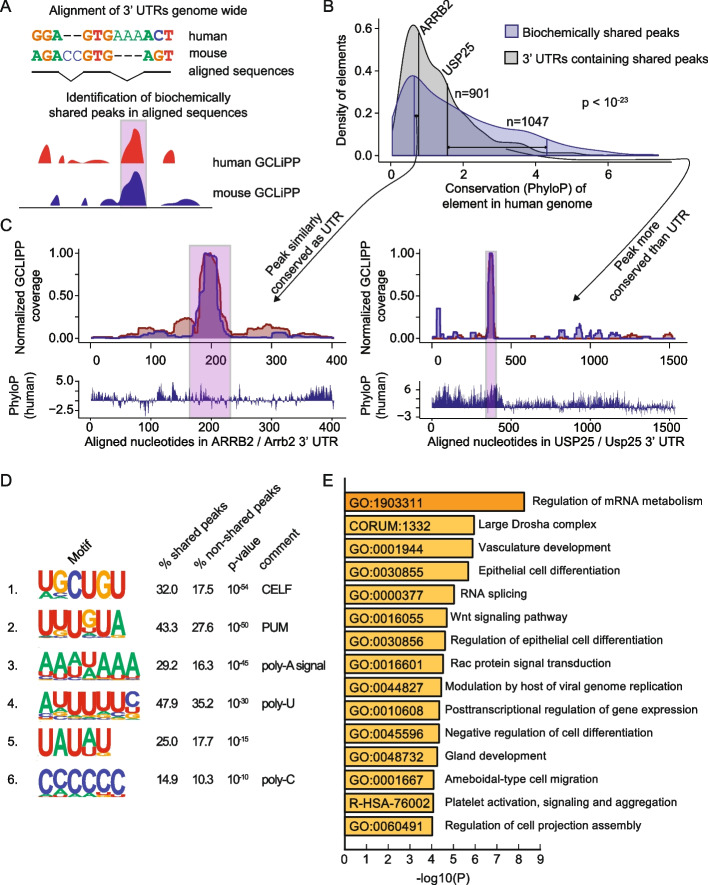
Comparison between mouse and human GCLiPP reveals principles of shared post-transcriptional regulation. **A** Schematic illustration of 3′ UTR alignment and biochemically shared GCLiPP peak calling. **B** Distribution of conservation across 100 vertebrates (PhyloP score) of regions in the human genome. Blue indicates biochemically shared peaks and gray indicates the 3′ UTRs of the transcripts that those peaks are contained within. For both peaks within *ARRB2* and *USP25*, their matched conservation of peak and UTR are indicated by connected vertical lines. **C** Human and mouse normalized GCLiPP density and conservation (PhyloP) across aligned nucleotides of the indicated 3′ UTRs. Biochemically shared peaks of GCLiPP read density are indicated in pink. **D** HOMER called motifs enriched in biochemically shared peaks. Percentages indicate the frequency of occurrence of the indicated motif in biochemically shared peaks versus non-shared background peaks. *P*-value indicates HOMER calculated *p*-value of enrichment. **E** Metascape called biological enrichment categories of genes containing biochemically shared peaks. The background set was all genes that contained peaks in both mouse and human GCLiPP datasets that did not contain a shared peak

Next, we sought to compare RBP occupancy in mouse and human T cells. To do so, we performed Clustal Omega sequence alignments of thousands of human 3′ UTRs and their corresponding sequences in the mouse genome, and then designed an algorithm to identify correlated peaks of normalized GCLiPP read density along the aligned nucleotides (Fig. 5A). Using this approach, we identified 1047 high-stringency biochemically shared GCLiPP peaks derived from 901 3′UTRs (Additional file 3: Table S3). As a class, biochemically shared peaks exhibited significantly higher sequence conservation than the full 3′ UTRs in which they reside (Fig. 5B). The highly conserved, biochemically shared peak in *USP25* exemplifies this general pattern (Fig. 5C, right panel). However, many biochemically shared peaks did not exhibit corresponding increases in local sequence conservation. For example, the *ARRB2* mRNA that encodes b-arrestin, another regulator of T cell migration in response to chemoattractant gradients [45], exhibited a common peak of RBP occupancy in Jurkat cells and primary mouse T cells that is roughly equally conserved as the rest of the 3′ UTR (Fig. 5C, left panel).

To examine which RBPs contributed to biochemically shared peaks more than other GCLiPP peaks, we used HOMER motif calling software [46] to identify enriched motifs. Strikingly, of the six linear sequence motifs present in > 10% of biochemically shared peaks with *p* ≤ 10^−10^, five resemble well-known regulatory sequences (Fig. 5D). The two most common appeared to represent canonical CELF [47] and PUM [48] binding motifs. Three other identified motifs corresponded to runs of homo-polymers: an A-rich motif that resembled the canonical PAS [49]; a poly-U containing motif similar to a sequence that has long been known to stabilize mRNAs [50] and a poly-C containing motif similar to the C-rich RNAs bound by poly-C binding proteins [51]. We used Metascape [52] to identify categories of biologically related genes enriched among mRNAs that contained biochemically shared GCLiPP peaks (Fig. 5E and Additional file 4: Table S4). Interestingly, 3 of the 5 most enriched categories were related to RNA regulation (“regulation of mRNA metabolism,” “large Drosha complex,” “RNA splicing”), with the broad category “post-transcriptional regulation of gene expression” also in the top 10. Thus, biochemically shared GCLiPP binding sites are generally more well conserved than their local sequence context, enriched for well-studied RBP binding motifs, and occur preferentially in genes that encode proteins involved in post-transcriptional gene regulation. Together, these observations suggest the presence of conserved autoregulatory gene expression networks.

### GCLiPP-guided CRISPR dissection of biochemically shared post-transcriptional cis-elements

**Fig. 6:**
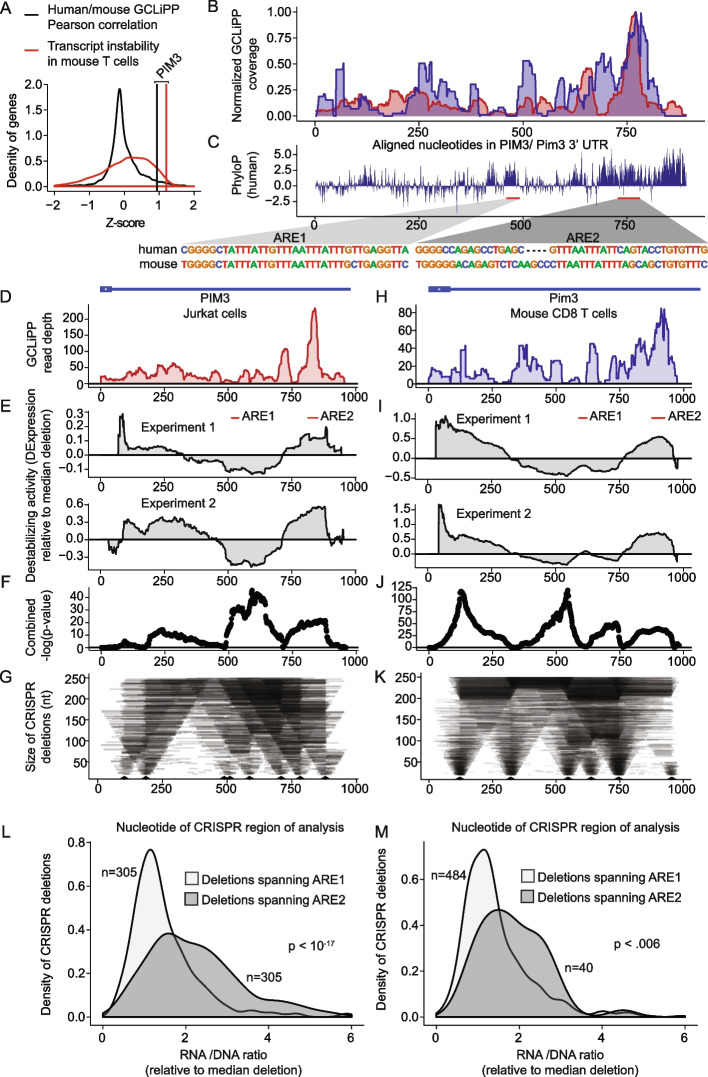
Biochemically and functionally shared post-transcriptional regulation of PIM3 in human and mouse cells. **A** *Z*-scores of Pearson correlation between mouse and human GCLiPP (black distribution) and transcript instability as measured by comparing transcript read abundance in untreated versus actinomycin D-treated mouse T cells (red distribution) for 7541 genes with matched data. Vertical lines indicate observations for *PIM3*. **B** Normalized human and mouse GCLiPP read density and **C** PhyloP across aligned nucleotides of *PIM3* 3′ UTR (as depicted in Fig. 5). Insets show sequences of putative regulatory elements. **D–G** Dissection of human *PIM3* 3′UTR in Jurkat T cells. **D** GCLiPP peaks aligned to schematic illustration of 3′UTR. **E** Change in expression along the 3′UTR relative to median expression of all possible deletions. Per-nucleotide effect score was calculated by comparing median normalized RNA/gDNA ratio for all shown deletions spanning a given nucleotide with median of all shown deletions. Experiment 1 and 2 are biological duplicates which were transfected with 80 or 120 μM of gRNAs respectively. Red bars indicate putative ARE-containing cis-regulatory elements. **F** Unadjusted − log10 *p*-values from Welch’s two sample *t*-test comparing all deletions spanning a nucleotide with all other deletions across both replicate experiments in **E**. **G** Size of deletions generated using CRISPR-Cas9. Arrow heads represent gRNA placement. **H–K** Dissection of mouse PIM3 3′UTR. Data are represented identically to human data, except that mouse primary CD8 T cells were used, and both mouse experiments 1 and 2 used a gRNA concentration of 80 μM. **L** Effect of deletions spanning putative ARE-containing cis-regulatory elements. The RNA/DNA ratio for mutants deleting ARE1 and ARE2 are shown in human Jurkat T cells. **M** Same as in **L** but using data from mouse primary T cells

We hypothesized that functionally conserved destabilizing cis-regulatory elements could be identified by examining biochemically shared GCLiPP peaks in 3′ UTRs of labile transcripts. To prioritize candidates, we computed Pearson correlation coefficients for the normalized GCLiPP profiles of 3′UTRs of genes expressed in both Jurkat cells and primary mouse T cells (Fig. 6A, black histogram) and examined transcript instability by RNAseq analysis of primary mouse T cells treated with actinomycin D (Fig. 6A, red histogram). The proto-oncogene *PIM3* emerged as an outstanding candidate with both strong interspecies GCLiPP correlation and very high transcript instability. Alignment of the GCLiPP profiles of human and mouse *PIM3* revealed a dominant shared peak of GCLiPP read density (Fig. 6B). This peak corresponded to a highly conserved region of the transcript that contains a G-quadruplex, followed by a putative AU-rich element (ARE) and a CELF binding motif (Fig. 6C). Another conserved region with G-quadruplex followed by a putative ARE appeared upstream of the biochemically share GCLiPP peak. We numbered these conserved regions ARE1 and ARE2 according to their order in the 3′UTR and hypothesized that ARE2 would exert greater cis-regulatory activity than ARE1, given its RBP occupancy in both species and the relative lack of occupancy in ARE1. To test this hypothesis, we performed CRISPR dissections of both the human and mouse *PIM3* 3′UTRs (Fig. 6 and Additional file 5: Table S5). These analyses produced largely concordant patterns of post-transcriptional cis-regulatory activity in the human (Fig. 6D–G) and mouse (Fig. 6H–K) 3′UTR, with the greatest significant destabilizing effect corresponding to the shared region of GCLiPP read intensity covering the ARE2 element. Consistent with this portrait of the entire 3′ UTR, when we filtered specifically for mutations that completely deleted either ARE1 or ARE2, we observed significantly greater expression of transcripts derived from cells with ARE2 deleted versus ARE1 (Fig. 6L, M). Thus, *PIM3* is a very unstable transcript with highly concordant RBP occupancy in human and mouse cells. Functional dissection of the post-transcriptional regulatory landscape of this gene revealed that this biochemical concordance between mouse and human cells is mirrored at a functional level, with the most highly occupied region indicated by GCLiPP read density corresponding to the most destabilizing region of the 3′ UTR.

### GCLiPP-guided functional analysis of autoimmune disease-associated SNPs

**Fig. 7:**
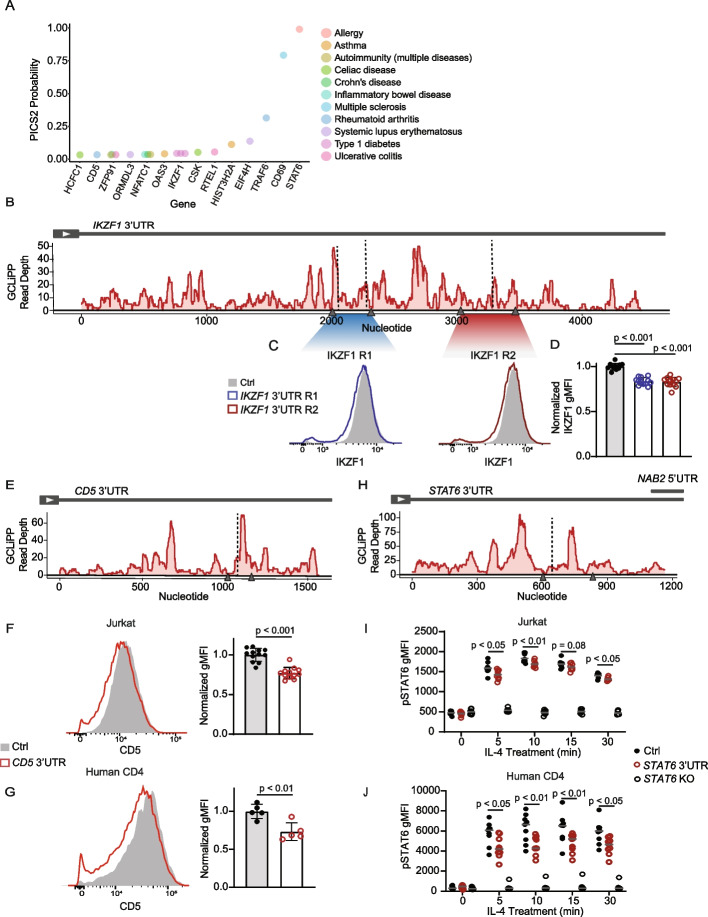
GCLiPP and PICS2-identified probable causal SNPs guide dissection of cis-regulatory elements in 3′UTR. **A** Top 15 PICS2 SNPs within GCLiPP peaks with gene location (*x*-axis) and ranked by PICS2 probability score (*y*-axis). Diseases associated with SNPs are marked by color. **B** GCLiPP track of *IKZF1* 3′UTR. Arrow heads represent gRNA placement to delete two regions (R1 and R2). Vertical dotted line indicates variant location. **C** Representative IKZF1 protein expression detected by intracellular flow cytometry in Jurkat cells edited with non-targeting control gRNAs (Ctrl), or paired gRNAs targeting *IKZF1* 3′UTR R1 (blue) or IKZF1 3′UTR R2 deletion (red). **D** Normalized IKZF1 gMFI for 3 replicate CRISPR targetings from 2 independent experiments. **E** GCLiPP track of *CD5* 3′UTR in Jurkats, similar annotations as **B**. **F** CD5 expression in Jurkats cells and **G** primary human CD4 T cells targeted with non-targeting control gRNAs (Ctrl; gray) or *CD5* 3′UTR gRNAs to induce deletion (red). Histogram shows representative flow cytometry data (left) and normalized geometric mean fluorescence intensity (gMFI) for **F** 3 replicate CRISPR targeting in 3 independent experiments for Jurkats and **G** 5 replicates of individuals or pooled individuals from 2 independent experiments for primary human T cells. **H** GCLiPP track of *STAT6* 3′UTR in Jurkats. **I** pSTAT6 gMFI of Jurkat cells or **J** primary human CD4 T cells polarized to Th2 cells targeted with non-targeting control (Ctrl), *STAT6* coding region dual gRNAs (*STAT6* KO) or *STAT6* 3′UTR paired gRNAs following treatment with IL-4 for 0, 5, 10, 15, or 30 min. Data are shown for **I** 2–3 replicate CRISPR targetings from 3 independent experiments for Jurkats and **J** 9 individuals or pooled individuals from 3 independent experiments for primary human CD4 T cells

We reason that RBP occupancy maps could be used to guide functional annotation of sequence variants that lie within RNA in cis-regulatory elements. To test this possibility, we intersected our Jurkat GCLiPP peaks with probable casual single-nucleotide polymorphisms (SNPs) associated with human immune-mediated diseases. A previously developed algorithm, Probabilistic Identification of Casual SNPs (PICS) [14] identified candidate causal SNPs through fine-mapping that were linked to immune-mediated diseases. PICS2 [53] has expanded that list to include variants identified with more recently collected GWAS data. Within these variants, we identified 63 SNPs that appear within a GCLiPP peak in a 3′UTR in Jurkat cells (Additional file 6: Table S6). These variants were associated with a variety of immune-mediated disorders and appeared in a variety of genes that are expressed in T cells (Fig. 7A). To test whether disease-associated probable causal variants overlapping GCLiPP peaks mark functional RNA cis-regulatory elements, we deleted 4 individual RBP binding sites in the 3′UTRs of 3 distinct immunologically important genes using a dual guide RNA (gRNA) CRISPR-Cas9 editing approach.

Ikaros family zinc finger 1 (IKZF1) is a pleiotropic transcriptional factor involved in lymphocyte differentiation [54]. Its 3′UTR contains 3 probably causal SNPs associated with type 1 diabetes (Fig. 7B). We generated two separate deletions using paired gRNAs (Fig. 7B, gray arrow heads) containing these SNPs and observed decreased IKZF1 protein expression compared to control cells in Jurkats (Fig. 7C, D), suggesting presence of a cis-regulatory element in the 3′UTR.

Intersection of GCLiPP and PICS2 data also revealed a probable causal SNP associated with rheumatoid arthritis in the 3′UTR of *CD5* (Fig. 7E), which encodes an inhibitory receptor expressed on T cells [55]. Deletion of this region with paired gRNAs (at 50–60% editing efficiency; data not shown) decreased CD5 expression in Jurkats (Fig. 7F). To determine whether this effect is also observed in primary T cells, the same deletion was generated in human CD4 T cells and showed similar decreased in CD5 expression (Fig. 7G). Together, this suggests the presence of a cis-regulatory element in the 3′UTR of *CD5* that is conserved between Jurkat cell line and primary human T cells.

SNP rs1059513 in the 3′UTR of *STAT6* had a PICS2 probability score 0.985 for association with allergy, making it by far the most likely causal variant in the locus for this trait. STAT6 is an important signaling protein and transcription factor that is pivotal for mounting a type 2 inflammatory response. It is activated by Janus kinase (JAK)-mediated phosphorylation downstream of IL-4 and IL-13 signaling [56]. To determine whether the identified RBP binding site affected STAT6 expression and function, we used CRISPR-Cas9 to generate a small deletion (Fig. 7H) and treated the edited cells with IL-4 to measure phospho-STAT6 (pSTAT6). *STAT6* 3′UTR edited cells showed similar phosphorylation kinetics as control (Additional file 1: Fig. S5A), but overall decreased pSTAT6 expression compared to controls (Fig. 7I, Additional file 1: Fig. S5B) in Jurkats. The same deletion in primary CD4 T cells polarized toward Th2 cells also showed comparable phosphorylation kinetics as non-targeting control cells (Additional file 1: Fig. S5C) and decreased pSTAT6 expression during IL-4 treatment (Fig. 0.7 J, Additional file 1: Fig. S5D).

In summary, a GCLiPP-guided analysis of probable causal SNPs in 3′UTRs efficiently identified functional RNA cis-regulatory elements in human T cells that regulate protein expression. These findings demonstrate the utility of a transcriptome-wide profile of RBP occupancy in the T cell transcriptome.

## Discussion

Interconnected networks of RBPs and RNAs form a complex layer of post-transcriptional regulation that affects all biological processes. Understanding these networks remains one of the key challenges in deciphering how the genome encodes diverse cell identities and behaviors [14, 57]. Methods like DNase I hypersensitivity and ATAC-seq that query regulatory element accessibility and occupancy without prior knowledge of their protein-binding partners have proven themselves as powerful techniques for the systematic mapping of *cis*-regulatory sequences in DNA [58, 59]. Their development has allowed for comparisons in the regulatory structure of diverse cell types [60] and for functional analysis of genetic variants [14]. Large-scale eCLIP analyses of individual RBPs have begun the intensive process of documenting RBP binding sites in the transcriptome of a few model cell types, providing a useful repository of RNA regulatory data [19, 61, 62]. Here, we describe GCLiPP, an optimized method for global RBP occupancy mapping with methodologic and performance similarities to eCLIP. We generated and validated a RBP binding map of the transcriptome in T cells and used it as a guide to identify cis-regulatory elements in 3′UTRs. As ATAC-seq has been used to define global regulatory elements involved in transcription, we demonstrated the use of GCLiPP to discover RNA regulatory elements that mediate post-transcriptional gene regulation.

Our data demonstrate that GCLiPP maps RBP occupancy at a higher resolution than what has been achieved with organic phase separation techniques. This feature, together with its technical similarity with RBP-specific eCLIP, make GCLiPP a particularly valuable tool for the identification and functional analysis of RNA cis-regulatory elements. The preferential capture of polyadenylated transcripts is both a feature and a limitation of GCLiPP. Methods using proximity-based CLIP [61], locked nucleic acid (LNA) capture probes [18], and organic phase separation [15, 16] more broadly represent non-polyadenylated noncoding RNAs. Similar to these other techniques, GCLiPP relies on UV crosslinking to isolate RBPs, likely preferentially capturing ssRNA while under-sampling double-stranded cis-regulatory elements. In the future, GCLiPP could be modified to include LNA probes to diversify the types of transcripts captured, and improved with a ribosomal depletion step to limit rRNA in the sample.

Dissection of the human *PIM3* and mouse *Pim3* 3′UTRs demonstrated the utility of GCLiPP for decoding biochemically shared and functionally conserved post-transcriptional regulation. The PIM family of serine/threonine kinases exert profound regulatory effects on MYC activity, cap-dependent translation independent of mTOR, and BAD-mediated antagonism of apoptosis [62]. Post-transcriptional regulation of PIM kinases is important, as proviral integrations in the *Pim1* 3′ UTR are highly oncogenic [63]. *Pim3* mRNA was abundant but highly labile in T cells, with a turnover rate in the top 2% of expressed mRNAs. PIM family members contain multiple ARE-like repeats of AUUU(A), but the specific sequences responsible for rapid mRNA decay have not been described and cannot be predicted from the primary sequence alone. The *PIM3* 3′UTR contains two phylogenetically conserved regions with very similar predicted ARE sequences. Of these regions, we predicted that greater regulatory activity would be exerted by the region with GCLiPP evidence for RBP occupancy in both human and mouse cells*.* CRISPR dissection bore out this prediction in both species. The inactive conserved region may be structurally inaccessible to RBP occupancy, or it may be occupied and exert regulatory activity only in other cell types or signaling conditions.

Targeted dissection of GCLiPP-identified RBP binding regions within 3′UTRs of immunologically relevant genes also led to discovery of cis-regulatory regions that modulate protein expression. Decreased expression of both CD5 and IKZF1 after deletion of the targeted regions suggests the presence of a post-transcriptional stabilizing or translational element. Lower levels of pSTAT6 similarly indicate stabilizing activity in *STAT6* 3′UTR. Our data uncovered conserved regulatory activity in the dissected 3′UTRs in both Jurkats and primary human T cells, demonstrating the utility of using Jurkat RBP binding data to guide discovery of post-transcriptional elements for shared expressed genes in primary T cells.

The mechanism by which these elements affect protein expression, and their role in regulating T cell biology is not yet well-defined. However, quantitative changes in CD5 and IKZF1 expression are expected to alter T cell activation and differentiation, respectively [54, 55, 64]. STAT6 plays clear mechanistic roles in allergy and asthma, and a recent study showed that altered STAT6 expression due to rare germline gain of function promoter mutations cause severe allergic disorders [56]. Mechanistic investigation is warranted to understand how the RBP-occupied region containing a highly probable causal SNP for allergy regulates STAT6 expression and T cell biology in the context of allergic responses. Together, these targeted dissections further highlight the utility of unbiased high-resolution biochemical determination of RBP occupancy for annotating the regulatory transcriptome in conjunction with genetic data.

Systematic comparison with eCLIP data for 87 individual RBPs [32] indicated that GCLiPP roughly represented a weighted average of all potential eCLIP experiments for cytosolic RBPs. GCLiPP peaks overlapped eCLIP peaks at a frequency much greater than would be expected by chance, even though different cell types were used for the GCLiPP and eCLIP experiments. These findings are consistent with the prior observation that binding sites for individual proteins detected by eCLIP generally differ little between cell types with different tissue origin [19]. Nevertheless, the precise profiles of RBP occupancy and regulation of individual transcripts may be subject to cell type and context-dependent differences in RBP expression, binding activity, and site accessibility. Overall GCLiPP read density correlated with eCLIP read density in a manner that corresponded with the relative abundance of a given RBP in purified cellular mRNPs [21]. Still, the eCLIP peaks for some low abundance RBPs were significantly enriched in GCLiPP profiles. The strongest correlations were observed for abundant cytosolic RBPs, and the correspondence between eCLIP and GCLiPP was only apparent for cytosolic, but not non-cytosolic RBPs. This result was expected since the GCLiPP protocol selectively enriches for cytosolic polyadenylated RNA. GCLiPP could be modified to intentionally enrich for nuclear RBPs to examine the regulatory landscape of mRNA biogenesis.

We leveraged the matched datasets from similar cell types expressing many shared transcripts to perform a cross-species comparison of the post-transcriptional regulatory landscape. As might be expected, the sequences of 3′ UTR regions that appeared as peaks of RBP occupancy in both species were in general more conserved than the full-length 3′ UTRs in which they occurred. These biochemically shared peaks were enriched in well-known RBP-binding cis-regulatory sequences including PUM motifs, CELF motifs, and canonical polyadenylation signals. We also found clear biochemically shared peaks with relatively poor sequence conservation. These regions retain RBP occupancy despite an evident lack of strong selective pressure on their primary sequence, perhaps due to highly degenerate and/or structural determinants of RBP occupancy. RNAs with conserved structure and RBP binding but poorly conserved primary sequence have been reported before, and they are enriched in gene regulatory regions [65, 66]. Finally, we noted that transcripts with biochemically shared peaks tended to encode proteins that were themselves involved in post-transcriptional gene regulation. This pattern is consistent with previous suggestions that autoregulatory or multi-component feedback loops may be a conserved mode of post-transcriptional gene regulation [67].

## Conclusion

The GCLiPP datasets reported here provide a rich resource for the annotation and experimental dissection of cis-regulatory function in mRNAs. GCLiPP detected RBP occupancy at many known cis-regulatory regions, including canonical polyadenylation signals and elements that control mRNA localization, translation, and stability, and provide a biochemical correlate of functional activity. Our method generated higher-resolution mapping of RBP binding sites compared to phase separation biochemical approaches, similar to ENCORE. These data are provided to the scientific community for browsing and mining in a readily accessible form online. Combining GCLiPP with unbiased biochemical assays, genetic analyses and other datasets probing RNA regulatory circuits will yield a roadmap for the dissection of post-transcriptional regulatory networks and hypothesis generation of multi-omics studies.

## Methods

### Cells

Primary CD4^+^ and CD8^+^ mouse T cells were isolated from C57BL/6 J mouse peripheral lymph nodes and spleen using positive and negative selection Dynabeads, respectively, according to the manufacturer’s instructions (Invitrogen). All mice were housed and bred in specific pathogen-free conditions in the Animal Barrier Facility at the University of California, San Francisco. Animal experiments were approved by the Institutional Animal Care and Use Committee of the University of California, San Francisco. Cells were stimulated with immobilized biotinylated anti-CD3 (0.25 mg/mL, BioXcell, clone 2C11) and anti-CD28 (1 mg/mL, BioXcell, clone 37.51) bound to Corning 10-cm cell culture dishes coated with Neutravidin (Thermo) at 10 mg/mL in PBS for 3 h at 37 °C. Cells were left on stimulation for 3 days before being transferred to non-coated dishes in T cell medium [68] supplemented with recombinant human IL-2 (20 U/mL, NCI). Th2 cell cultures were also supplemented with murine IL-4 (100 U/mL) and anti-mouse IFN-γ (10 µg/mL). CD8 T cell cultures were also supplemented with 10 ng/mL recombinant murine IL-12 (10 ng/mL). For re-stimulation, cells were treated with 20 nM phorbol 12-myristate 13-acetate (PMA) and 1 µM ionomycin (Sigma-Aldrich) for 4 h before harvest.

Peripheral blood mononuclear cells (PBMCs) were isolated from anonymous donors through Ficoll-Paque Plus centrifugation gradient (Cytiva). CD4 T cells were isolated from PBMCs using EasySep Human CD4 + Isolation Kit according to the manufacturer’s protocol (StemCell Technologies). Cells were stimulated on plates coated with anti-CD3 (1 μg/ml, UCSF Monoclonal Antibody Core; clone OKT-3) and anti-CD28 (2 μg/ml, Miltenyi Biotec; clone 15E8). After 2 days of stimulation, cells were electroporated to incorporate CRISPR-Cas9 RNPs and placed back on anti-CD3- and anti-CD28-coated plates for 1 day. Cells were then rested in T cell media supplemented with recombinant human IL-2 (20 U/mL, NCI). For Th2 polarizing conditions, cultures were supplemented with human recombinant IL-4 (12.5 ng/mL, R&D Systems) and human anti-IFN- γ (10 μg/ml, Invitrogen, clone NIB42) during stimulation and only with anti-IFN- γ (5 μg/ml) during rest. Protein readout for CD5 was conducted 4 days after electroporation and 6 days for pSTAT6. T cell media consisted of RPMI-1640 supplemented with 10% fetal bovine serum (FBS) (Omega), L-glutamine, penicillin, streptomycin, sodium pyruvate, β-mercaptoethanol, and HEPES. Jurkat cells were grown in RPMI supplemented with FBS, L-glutamine, penicillin, and streptomycin.

### Measurement of mRNA decay

Cells were stimulated with PMA and Ionomycin for 4 h and then additionally treated with actinomycin D (Sigma-Aldrich) at 5 µg/mL for an additional 0, 1, 2, or 4 h. After treatment, cells were lysed with Trizol LS (Life Technologies) and processed with Direct-zol™ 96-well RNA (Zymogen). RNA was quantified with an ND-1000 spectrophotometer (NanoDrop) and reverse transcribed with SuperScript III First Strand Synthesis Kit (Invitrogen).

### GCLiPP and RNAseq

 ~ 100 × 10^6^ mouse T cells cultured from 3 mice or ~ 100 × 10^6^ Jurkat T cells were washed and resuspended in ice-cold PBS and UV irradiated with a 254-nm UV crosslinker (Stratagene) in three doses of 4000, 2000, and 2000 mJ, swirling on ice between doses. Cells were pelleted and frozen at − 80 °C. Thawed pellets were rapidly resuspended in 400 µL PXL buffer without SDS (1 × PBS with 0.5% deoxycholate, 0.5% NP-40, Protease inhibitor cocktail) supplemented with 2000 U RNasin (Promega) and 10 U DNase (Invitrogen). Pellets were incubated at 37 °C with shaking for 10 min, before pelleting of nuclei and cell debris (17,000* g* for 5 min). Supernatants were biotinylated by mixing at room temperature for 30 min with 500 µL of 10 mM EZ-Link NHS- SS-Biotin (Thermo) and 100 µL of 1 M sodium bicarbonate. Supernatants were mixed with 1 mg of washed oligo-dT beads (New England Biolabs) at room temperature for 30 min and washed 3 times with magnetic separation. Oligo-dT selected RNA was eluted from beads by heating in poly-A elution buffer (New England Biolabs) at 65 °C with vigorous shaking for 10 min. An aliquot of eluted RNA was treated with proteinase K and saved for RNAseq analysis using Illumina TruSeq Stranded Total RNA Library Prep Kit according to the manufacturer’s instructions. Cells treated with actinomycin D as described above were also collected for RNAseq to generate transcriptome-wide measurements of transcript stability.

The remaining crosslinked, biotinylated mRNA-RBP complexes were captured on 250 µL of washed M-280 Streptavidin Dynabeads (Invitrogen) for 30 min at 4 °C with continuous rotation to mix. Beads were washed 3 times with PBS and resuspended in 40 µL of PBS containing 1000 U of RNase T1 (Thermo) for 1 min at room temperature. RNase activity was stopped by addition of concentrated (10% w/v) SDS to a final concentration of 1% SDS. Beads were washed successively in 1 × PXL buffer, 5 × PXL buffer, and twice in PBS. Twenty-four picomoles of 3′ radiolabeled RNA linker was ligated to RBP-bound RNA fragments by resuspending beads in 20 µL ligation buffer containing 10 U T4 RNA Ligase 1 (New England Biolabs) with 20% PEG 8000 at 37° for 3 h. Beads were washed 3 × with PBS and free 5′ RNA ends were phosphorylated with polynucleotide kinase (New England Biolabs). Beads were washed 3 × with PBS and resuspended in ligation buffer containing 10 U T4 RNA Ligase 1, 50 pmol of 5′ RNA linker, and 20% PEG 8000 and incubated at 15 °C overnight with intermittent mixing. Beads were again washed 3 times in PBS and linker-ligated RBP-binding fragments were eluted by treatment with proteinase K (Sigma-Aldrich) in 20 µL PBS with high-speed shaking at 55 °C. Beads and supernatant were mixed 1:1 with bromophenol blue formamide RNA gel loading dye (Thermo) and loaded onto a 15% TBE-Urea denaturing polyacrylamide gel (Bio-Rad). Ligated products with insert were visualized by autoradiography and compared to a control ligation (19 and 24 nt markers). Gel slices were crushed and soaked in gel diffusion buffer (0.5 M ammonium acetate; 10 mM magnesium acetate; 1 mM EDTA, pH 8.0; 0.1% SDS) at 37 °C for 30 min with high-speed shaking, ethanol precipitated, and resuspended in 20 µL of RNase-free water. Ligated RNAs were reverse transcribed with Superscript III reverse transcriptase (Invitrogen) and amplified with Q5 polymerase (New England Biolabs). PCR was monitored using a real-time PCR thermal cycler and amplification was discontinued when it ceased to amplify linearly. PCR products were run on a 10% TBE polyacrylamide gel (Bio-Rad), size selected for an amplicon with the predicted 20–50 bp insert size to exclude linker dimers, and purified from the gel (Qiagen). Cleaned up library DNA was quantified on an Agilent 2100 Bioanalyzer using the High Sensitivity DNA Kit before being sequenced. All GCLiPP and RNAseq sequencing runs were carried out on an Illumina HiSeq 2500 sequencer.

### GCLiPP and RNAseq bioinformatics analysis pipeline

FastQ files were de-multiplexed and trimmed of adapters. Each experiment was performed on three technical replicates per condition (resting and stimulated) per experiment. Cloning replicates and experiments were pooled in subsequent analyses. Jurkat and mouse T cell trimmed sequence reads were aligned to the hg38 human or mm10 mouse genome assembly using bowtie2, respectively. After alignment, PCR amplification artifacts were removed by de-duplication using the 2-nt random sequence at the 5′ end of the 3′ linker using a custom script that counted only a single read containing a unique linker sequence and start and end position of alignment per sequenced sample. Peaks of GCLiPP read density were called by convolving a normal distribution against a sliding window of the observed read distribution with a custom script (utr_peak_finder.pl). A 70-nucleotide window was analyzed centered on every nucleotide within the 3′ UTR. For each window, the observed distribution of read density was compared to a normal distribution of the same magnitude as the nucleotide in the center of the window. The Pearson correlation coefficient was computed for each nucleotide and peaks were defined as local maxima of goodness of fit between observed GCLiPP read density and the normal distribution, requiring a read depth above 20% of the maximum read depth in the 3′ UTR global minimum of 10 reads. RNAseq reads were aligned using STAR Aligner (https://github.com/alexdobin/STAR) [69] to align against the mm10 genome, and gene expression data were calculated as fragments per kilobase per million reads. Source code for data visualization software Thagomizer can be found at https://github.com/sskhon-2014/Graphy.

### Comparison of GCLiPP to individual eCLIP datasets

eCLIP data [32] from K562 cell line were downloaded via the ENCODE data portal (http://www.encodeproject.org/). The first replicate set of bigwig files were downloaded for each RBP deposited online at the time of analysis (December 2017) (Additional file 7, Table S7) as well as CLIPper-called peaks for the same. To facilitate comparisons with GCLiPP, we called GCLiPP peaks in the Jurkat data using CLIPper [29] after re-aligning Jurkat GCLiPP reads to hg19. Correlation analysis was performed with a custom perl script that calculated the Spearman correlation for read depth at each nucleotide in the 3′ UTR of all genes that were expressed in each dataset (as determined by CLIP read depth). ~ 5000–15,000 expressed genes were included in the correlation analysis for each RBP. For comparison to mRNP abundancy, log10 RBP mass spectrometry spectra counts of HEK293 cells were utilized from [21]. To stratify RBPs by subcellular localization, data were taken from the COMPARTMENTS database, with RBPs with a localization score of 5 in the cytosol counted as cytosolic and lower counted as non-cytosolic [33]. All custom scripts are available at https://github.com/AnselLab/GCLiPP-Manuscript-scripts.

### RBP domain analysis

We called Jurkat GCLiPP peaks aligned to hg38 using CLIPper2.0 [29]. Each peak was resized to 200 bp and oriented at the original peak center. The 200 bp RNA sequence of each peak was analyzed using pf_fold method from ViennaRNA (RNAlib version 2.4.13) [30] to calculate base-pairing probability for each pair of nucleotides and presented as an average for all the identified RBP binding sites. The PTBP1 eCLIP dataset (hg38) from K562 cells was downloaded from ENCORE (GSM2424223) and processed in similar manner. The matrices in Fig. 2A and Fig. S1A are zoomed into the central 150-bp region.

We used available resting and activated Jurkat expression data [70] (GSE145453) to calculate read counts mapped to RBP domains using annotations from RBPDBv1.3 [71] as a reference. Proteomics data of RBPs expressed in human Th0 cells was obtained and identified as described [12]. RBPs that contained more than one annotated domain based on RBPDBv1.3 were considered as an individual count in each appropriate category.

### Conservation of RBP binding sites

To evaluate sequence conservation across various datasets, we performed CLIPper2.0 peak calling on sequencing data obtained through XRNAX [15] and OOPS [16]. The average PhyloP conservation score, obtained from UCSC genome browser as a bigwig of PhyloP scores of conservation 100 vertebrates, was calculated across all the sites within each method. This average was then standardized to contain a mean of 0 and a standard deviation of 1. Sequencing data for XRNAX (PRJEB26441; run accession ERR2537875) and OOPS (PRJEB26736; run accession SAMEA4663545, SAMEA4663546, SAMEA4663547, SAMEA4663548) was retrieved from EMBL-EBI ENA server and mapped to hg38 before CLIPper2.0 analysis. Specifically, our analysis used XRNAX data without ribosomal depletion and OOPS data performed using 150 mJ/cm^2^ crosslinking condition.

### Identifying differential RBP binding

We used DeepRNAreg (accompanying manuscript available upon request) to compare GCLiPP datasets from activated and resting Jurkat cells to obtain a list of genomic loci within 3′UTRs that were enriched in either condition, and assign a differential binding intensity (DBI) value to each site. This list of loci was intersected with all ENCORE eCLIP datasets for K562 cells to assign corresponding predicted RBPs for each identified binding region. For regions assigned to each RBP, we calculated the mean DBI for activated and resting Jurkat cells, and expressed the mean DBI fold change as the ratio of these means. Gene expression in activated and resting Jurkat cells was determined by calculating total read counts from Jurkat expression data [70] (GSE145453). Source code for DeepRNAreg is available at https://github.com/AnselLab/DeepRNA-Reg.

The same sets of regions differentially bound in activated or resting Jurkat cells were scored for the presence of consensus RBP recognition motifs within an 8-base pair window centered at the differential binding site. Enrichment of each binding motif within these regions was calculated against the background frequency of the same motif within the entire set of 3′ UTRs of genes bearing differentially bound regions. This analysis was performed for 119 RBPs that are represented in the oRNAment database of consensus binding sequences [34] and expressed in Jurkat cells [70].

### CRISPR editing

Guide RNA sequences were selected using the Benchling online CRISPR design tool (https://benchling.com/crispr) with guides selected to target genomic regions of GCLiPP read density. Synthetic crRNAs and tracrRNA (Dharmacon) were resuspended in water or 10 mM Tris–HCl Buffer pH 7.4 (Dharmacon) at 160 µM and allowed to hybridize at 1:1 ratio for 30 m at 37 °C. For CRISPR dissection experiments, all crRNAs were mixed at an equimolar ratio before annealing to tracrRNA. This annealed gRNA complex (80 µM) was then mixed 1:1 by volume with 40 µM *S. pyogenes* Cas9-NLS (University of California Berkeley QB3 Macrolab) to a final concentration of 20 µM Cas9 ribonucleotide complex (RNP). The complexed gRNA:Cas9 RNP and random 200 bp ssDNA (100 pmol, IDT) were nucleofected into primary mouse T cells (24 h after stimulation) or primary human T cells (48 h after stimulation) with the P3 Primary Cell 96-well Nucleofector™ Kit or into Jurkat cells with the SE Cell Line 96-well Nucleofector™ Kit using a 4-D Nucleofector following the manufacturer’s recommendations (Lonza). Cells were pipetted into pre-warmed media and then returned to CD3/CD28 stimulation for another 2 days for primary mouse T cells or 1 day for primary T cells and then expanded for an additional 3–5 days. Jurkat cells were expanded in resting conditions for 3–10 days after electroporation.

To validate deletion, gDNA was isolated from a portion of the edited cells using QuickExtract DNA Solution (Lucigen) following the manufacturer’s protocol. Edited regions were amplified through PCR using designed primers and MyTaq 2 × Red Mix (Bioline) and ran on 2% agarose gel. DNA bands were detected and quantified using Bio-Rad ChemiDoc.

### 3′ UTR dissection

3′ UTR dissection was performed as described [72]. Gene edited cells were harvested into Trizol reagent (Invitrogen) and total RNA was phase separated and purified from the aqueous phase using the Direct-zol RNA miniprep kit with on-column DNase treatment (Zymogen). Genomic DNA was extracted from the remaining organic phase by vigorous mixing with back extraction buffer (4 M guanidine thiocyanate, 50 mM sodium citrate, 1 M Tris base). cDNA was prepared with oligo-dT using the SuperScript III reverse transcription kit (Invitrogen). cDNA and genomic DNA were used as a template for PCR using MyTaq 2 × Red Mix (Bioline). To equilibrate the number of target molecules and number of PCR cycles between samples, we performed semi-quantitative PCR followed by agarose gel electrophoresis to determine a PCR cycle number where genomic DNA first showed visible bands. This cycle number was then used with a titration of cDNA concentrations. A concentration that amplified equivalently was selected for analysis by deep sequencing. To quantify relative RNA/DNA ratios, cDNA and genomic DNA amplicons were purified using a QIAquick PCR purification Kit (Qiagen) and quantified on an Agilent 2100 Bioanalyzer using the High Sensitivity DNA Kit (Agilent).

Amplicons were tagmented with the Nextera XT kit (Illumina) and sequenced on an Illumina 2500 HiSeq. Reads were aligned to a custom genome consisting of the targeted PCR amplicon using STAR aligner and mutations were scored using an awk script (https://github.com/alexdobin/STAR/blob/master/extras/scripts/sjFromSAMcollapseUandM.awk). RNA/DNA read ratios were calculated for all mutations over 20 nucleotides long and less than 250 nucleotides long, and relative expression was quantified as the median normalized RNA/DNA ratio for this subset of mutations. Mutations had to have at least 10 reads in both the RNA and gDNA amplicons and mutations with an RNA/DNA ratio of greater than 10 were excluded as outliers. Effect sizes for each nucleotide of the amplicon in each experiment were computed by comparing this median normalized RNA/DNA ratio for all mutations spanning a given nucleotide to all other mutations. Combined *p*-values were calculated using Welch’s two sample *t*-test comparing all mutations spanning a given nucleotide with all other mutations.

### Shared peak calling, motif analysis and icSHAPE and phylogenetic analyses

3′ UTR alignments of mouse and human were performed by downloading hg38 RefSeq 3′ UTRs from UCSC genome browser (http://genome.ucsc.edu), identifying syntenic regions of the mouse genome in mm10 with the KentUtils liftOver program (https://github.com/ucscGenomeBrowser/kent) and aligning UTRs with Clustal Omega (http://www.ebi.ac.uk/Tools/msa/clustalo/) [73]. Alignments were programmatically performed for all human 3′ UTRs with a custom perl script (get_alignment_from_fasta.pl). Biochemically shared peaks were called by the following algorithm (implemented in conserved_peak_finder.pl). This algorithm normalizes GCLiPP read density (i.e., the fraction of the maximal read depth within that 3′ UTR) at each position and calculates the correlation between mouse and human normalized signal. To favor regions with a clear local peak of GCLiPP read density, the algorithm further calculates the correlation between the observed data and a normal distribution centered at the point being examined in both the mouse and human data tracks. These three Spearman correlations were added together to calculate a numerical score, and shared peaks were defined as local maxima of these scores. To identify high-stringency peaks, peaks were only accepted if they (1) had a correlation of > 0.75 between mouse and human, (2) had a peak that had a read density of > 0.5 of the maximum read density within that 3′ UTR in one data track (mouse or human) and > 0.2 in the other, and (3) had > 10 reads at that location in both mouse and human datasets. Biological enrichment of genes with shared peaks was calculated using the Metascape [52] online interface (http://metascape.org) using the default settings, with the exception that a background set of genes was included in the analysis, specifically all genes that contain a called GCLiPP peak in both human and mouse datasets that do not contain a biochemically shared peak.

For motif calling, HOMER [46] was used in RNA mode with the “noweight” option to turn off GC correction to search for motifs of width 5, 6, or 7 nucleotides, with otherwise default parameters. The positive sequence set was the mouse and human sequences of the biochemically shared GCLiPP peaks, the negative sequence set was all other GCLiPP-called peaks from Jurkat and mouse T cells that were not shared across species. For icSHAPE, we used a published bigwig file of locally normalized icSHAPE signal intensity generated in mouse ES cell [44]. Conservation of loci in the mouse and human genomes were obtained from the UCSC genome browser as a bigwig of PhyloP scores of conservation across 60 placental mammals (mouse) and 100 vertebrates (human) (http://hgdownload.cse.ucsc.edu/goldenpath/mm10/phyloP60way/, http://hgdownload.cse.ucsc.edu/goldenpath/hg38/phyloP100way/).

### Mapping SNPs within GCLiPP peaks

We intersected our list of 3′UTR RBP peaks, determined using our peak calling algorithm, with a curated list of predicted disease causal SNPs [53] to identify SNPs within predicted RBP binding regions. We limited our analysis to SNPs located in the 3′UTR of genes that contained at least 1 GCLiPP peak. Specific regions in the 3′UTR of *CD5*, *IKZF1*, and *STAT6* were deleted in resting Jurkats using CRISPR-Cas9 RNPs as previously mentioned. Protein expression of the edited genes was measured by flow cytometry 3–5 days after nucleofection.

### Flow cytometry

Cells were stained with Live/Dead eFluor780 (Invitrogen) and anti-human CD5 (UCHT2) or intracellularly with anti-human IKZF1 (R32-1149) using the Foxp3 Transcription Factor Staining Kit (eBioscience). For pSTAT6 expression, Jurkat cells or primary human T cells were treated with recombinant human IL-4 (12.5 ng/mL; R&D Systems) for 0, 5, 10, 15, or 30 min, immediately fixed with 1.5% PFA for 10 min and permeabilized with ice-cold methanol for 15 min before staining with pSTAT6 (A15137E) for 1 h at room temperature. Primary T cells were additionally stained with anti-human CD4 (OKT4) and anti-human CD8 (HIT8a). Cells were analyzed on LSRII and FACSAria cytometers. GraphPad Prism was used for data visualization and for Mann–Whitney two-tailed *t-*test.

### Oligonucleotide and primer sequences

GCLiPP 3′ RNA linker: 5′-NNGUGUCUUUACACAGCUACGGCGUCG-3′

GCLiPP 5′ RNA linker: 5′-CGACCAGCAUCGACUCAGAAG-3′

GCLiPP Reverse transcription primer: 5′-CAAGCAGAAGACGGCATACGAGATNNNNNNCGCTAGTGACTGGAGTTCAGACGTGTGCTCTTCCGATCCGACGCCGTAGCTGTGTAAA-3′ (NNNNNN is barcode for demultiplexing).

GCLiPP 3′ PCR primer: 5′-CAAGCAGAAGACGGCATACGAGAT-3′

GCLiPP 5′ PCR primer: 5′-AATGATACGGCGACCACCGAGATCTACACTGGTACTCCGACCAGCATCGACTCAGAAG-3′

Read1seq sequencing primer for GCLiPP: 5′-ACACTGGTACTCCGACCAGCATCGACTCAGAAG-3′Index sequencer primer for GCLiPP: 5′-GATCGGAAGAGCACACGTCTGAACTCCAGTCAC-3′

PIM3 (human) gRNA1: TGTGCAGGCATCGCAGATGG

PIM3 (human) gRNA2: GACTTTGTACAGTCTGCTTG

PIM3 (human) gRNA3: GTGGCTAACTTAAGGGGAGT

PIM3 (human) gRNA4: AAACAATAAATAGCCCCGGT

PIM3 (human) gRNA5: TTGAGAAAACCAAGTCCCGC

PIM3 (human) gRNA6: CAGGAGGAGACGGCCCACGC

PIM3 (human) gRNA7: TTTATGGTGTGACCCCCTGG

PIM3 (human) gRNA8: CCAAGCCCCAGGGGACAGTG

Pim3 (mouse) gRNA1: GTTCAATTCTGGGAGAGCGC

Pim3 (mouse) gRNA2 CTGGTTCAAGTATCCACCCA

Pim3 (mouse) gRNA3: CCATAAATAAGAGACCGTGG

Pim3 (mouse) gRNA4: GCTTCCTCCCGCAAACACGG

Pim3 (mouse) gRNA5: CTGGTGTGACTAAGCATCAG

Pim3 (mouse) gRNA6: TGGAGAAGGTGGTTGCTTGG

Primers

PIM3 F (human): TCCAGCAGCGAGAGCTTGTGAGGAG

PIM3 R(human): TGATCTCCAGACATCTCACTTTTGAACTG

PIM3 R2(human): TGAGATAGGTGCCTCACTGATTAAGCATTGGTGATCTCCAGACATCTCACTTTTGAACTG

Pim3 F (mouse): GCGTTCCAGAGAACTGTGACCTTCG

Pim3 R (mouse): TATGATCTTCAGACATTTCACACTTTTG

CD5 gRNA1: GGAGCCTCGGGTCTGATCAA

CD5 gRNA2: GCTCTTCCAGACTTATTATG

IKZF1 R1 gRNA1: AAGGCTGACTTGTGTTCATG

IKZF1 R1 gRNA2: GCAACAAACTGACTCTAAGA

IKZF1 R2 gRNA1: TTATCATTGCATATCAGCAA

IKZF1 R2 gRNA2: ACATAATGCTTTTGGTGCGA

STAT6 gRNA1: GGGGTTAGCATATGTCAGAG

STAT6 gRNA2: CCAAATTCCTGTTAGCCAGG

STAT6 KO gRNA1: TCATAAGAAGGCACCATGGT

STAT6 KO gRNA2: CTGGATCCTCTTCAGCACTA

### Supplementary Information

**Additional file 1:Fig S1.** Comparison GCLiPP with eCLIP datasets. **Fig S2.** Overlap of GCLiPP peaks and cytosolic RBP eCLIP peaks. **Fig S3.** GCLiPP read coverage in primary mouse T cells. **Fig S4.** GCLiPP detects RBP binding of canonical polyadenylation signal. **Fig S5.** STAT6 expression in 3’UTR edited Jurkat cells and primary and primary human CD4 T cells.

**Additional file 2:Table S1.** Differential binding sites detected by DeepRNAReg enriched in stimulated Jurkats compared to unstimulated conditions. **Table S2.** Differential binding sites detected by DeepRNAReg enriched in unstimulated Jurkats compared to stimulated conditions.

**Additional file 3:Table S3.** Biochemically shared peaks in 3’UTRs between human Jurkat T cells and primary mouse T cells.

**Additional file 4:Table S4.** Genes with biochemically shared GCLiPP peaks in 3’UTRs identified through Metascape.

**Additional file 5:Table S5.** Fragments of human PIM3 and mouse Pim3 3’UTR generated from pooled CRISPR-Cas9 dissection.

**Additional file 6:Table S6.** Probable causal disease-associated SNPs identified by PICS2 that occur within GCLiPP-called RBP binding peaks.

**Additional file 7:Table S7.** List of RBP eCLIP datasets.

**Additional file 8.** Review history.

## Data Availability

The source code used in this manuscript is published in a freely accessible computational notebook under a Creative Commons Attribution 4.0 International license on Github and Zenodo [74, 75]. GCLiPP datasets are available from Gene Expression Omnibus (GEO), accessions GSE94554 and GSE115886 [76, 77]. Previously published Jurkat cell gene expression datasets are also available from GEO, accession GSE145453 [78]. XRNAX and OOPS data were downloaded from the European Nucleotide Archive (ENA), Projects PRJEB26441 (XRNA; sample accessions SAMEA4613241 and SAMEA4613244) [79] and PRJEB26736 (OOPS; sample accessions SAMEA4663545, SAMEA4663546, SAMEA4663547, SAMEA4663548) [80]. ENCORE eCLIP Peak Sets called by the algorithm CLIPper as TXT files in BED format from the ENCODE portal [81] (https://www.encodeproject.org/) were downloaded for datasets with the following GEO identifiers: GSM2424020, GSM2423898, GSM2423163, GSM2423828, GSM2423628, GSM2424038, GSM2424172, GSM2424114, GSM2424262, GSM2423694, GSM2424161, GSM2424183, GSM2423620, GSM2423807, GSM2424043, GSM2423297, GSM2424104, GSM2422882, GSM2424102, GSM2423325, GSM2424240, GSM2423957, GSM2423193, GSM2423213, GSM2423285, GSM2423796, GSM2423906, GSM2423711, GSM2423097, GSM2423241, GSM2423451, GSM2423602, GSM2423691, GSM2424216, GSM2422944, GSM2423480, GSM2423763, GSM2423478, GSM2424110, GSM2423509, GSM2424212, GSM2422904, GSM2423289, GSM2423152, GSM2423550, GSM2424058, GSM2424074, GSM2422967, GSM2423143, GSM2423630, GSM2424223, GSM2423824, GSM2423270, GSM2423381, GSM2423925, GSM2423137, GSM2423274, GSM2423562, GSM2423306, GSM2423243, GSM2424180, GSM2422937, GSM2423049, GSM2423071, GSM2423237, GSM2423548, GSM2422873, GSM2423821, GSM2423064, GSM2423475, GSM2423524, GSM2423683, GSM2423707, GSM2423584, GSM2422935, GSM2423379, GSM2423634, GSM2424062, GSM2424118, GSM2423357, GSM2423505, GSM2423222, GSM2423815, GSM2423618, GSM2424076, GSM2423817, GSM2423826.
