# Supplementary material for: GCLiPP: global crosslinking and protein purification method for constructing high-resolution occupancy maps for RNA binding proteins
Source: Genome Biol. 2023 Dec 7;24:281. doi: 10.1186/s13059-023-03125-2 (PMC10701951; doi:10.1186/s13059-023-03125-2)
Supplement: Supplementary file 1 — Additional file 1:Fig S1. Comparison GCLiPP with eCLIP datasets. Fig S2. Overlap of GCLiPP peaks and cytosolic RBP eCLIP peaks. Fig S3. GCLiPP read coverage in primary mouse T cells. Fig S4. GCLiPP detects RBP binding of canonical polyadenylation signal. Fig S5. STAT6 expression in 3’UTR edited Jurkat cells and primary and primary human CD4 T cells. [file 13059_2023_3125_MOESM1_ESM.pdf]

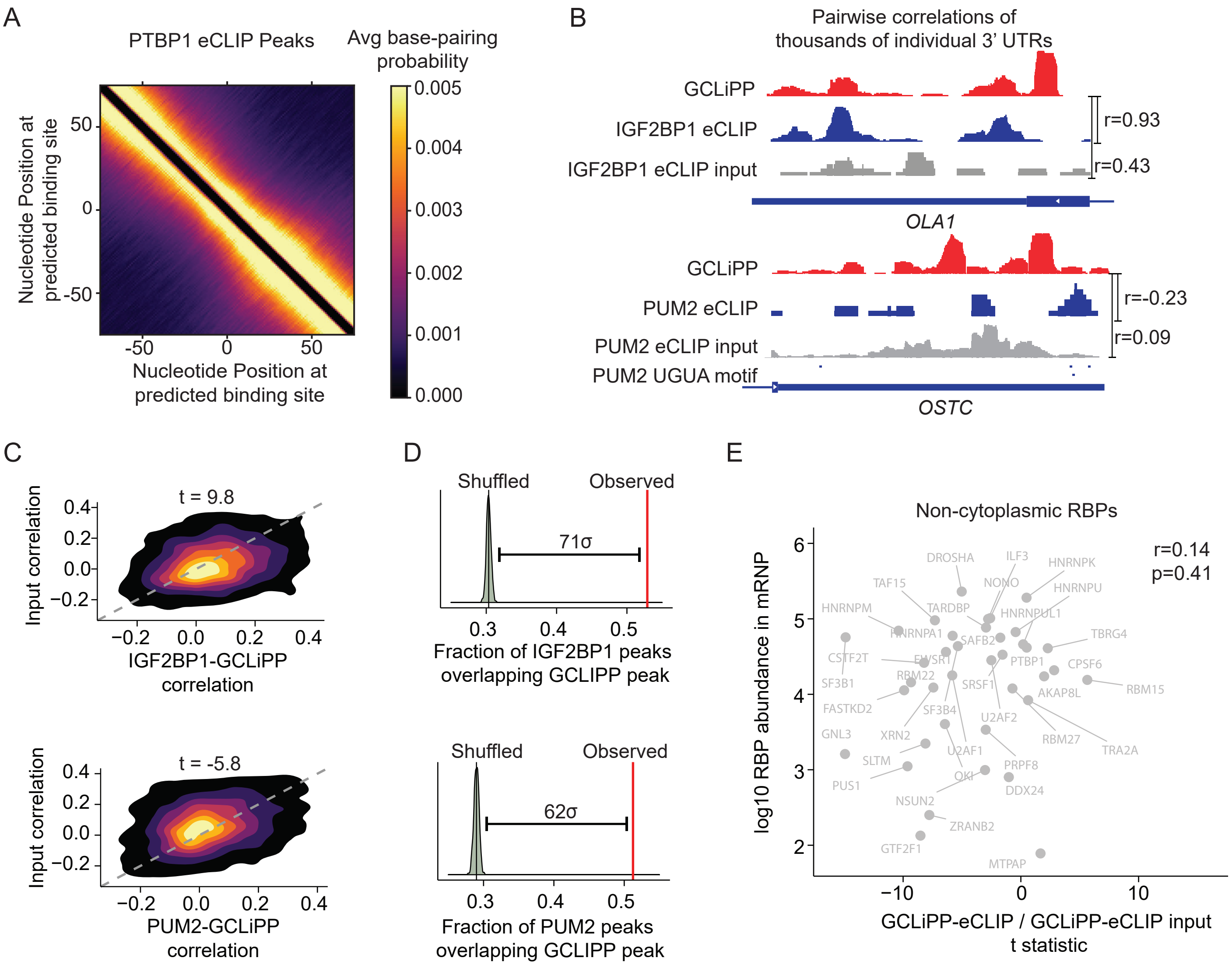

Fig S1. Comparison GCLiPP with eCLIP datasets.
(A) Average base-pairing probability for all PTBP1 binding sites captured through eCLIP in K562 cells. Peaks were called using CLIPper2.0 and matrix is shown as a heatmap as Fig. 2A. **(B)** Snapshots of individual 3'UTRs showing correlation between GCLiPP and IGFBP2 (top) and PUM2 (bottom) eCLIP datasets. GCLiPP shown in red, eCLIP in blue and matched control input samples in gray. r indicates Pearson correlation between pairs of normalized read density at a given nucleotide for the indicated comparison. **(C)** 2D density plots showing matched correlations between GCLiPP and eCLIP for indicated RBP (X-axis), and GCLiPP and control input sample (Y-axis) for individual 3'UTRs for all expressed genes in GCLiPP and eCLIP datasets. Paired t-test was used for the correlations. **(D)** Overlap of CLIPper called peaks in 3'UTRs of GCLiPP and eCLIP for indicated RBP. Red lines indicated observed overlap of GCLiPP peaks with eCLIP peaks. Green distribution represents bootstrapped expected overlap. Computed shuffling was conducted as described in Fig. 2F. **(E)** Correlation of eCLIP-GCLiPP paired t-tests from (B) and RBP abundance in mRNPs not localized in the cytosol (<5 cytosolic according to COMPARTMENTS).

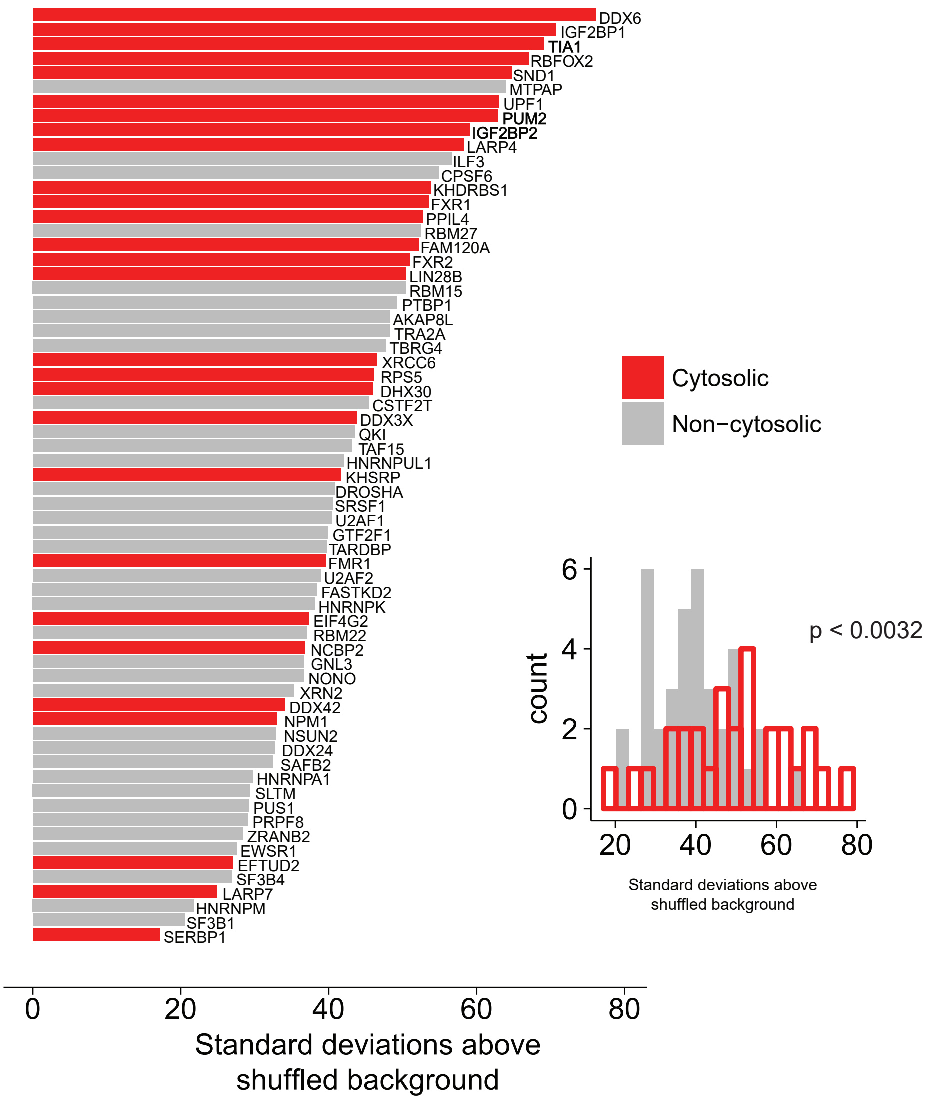

Fig S2. Overlap of GCLiPP peaks and cytosolic RBP eCLIP peaks. Overlap of CLIPper called peaks in 3' UTRs in GCLiPP and eCLIP as in Fig. 2E. The number of standard deviations between the observed fraction of eCLIP peaks that overlap with GCLiPP peaks above a background of 500 shuffled versions of the eCLIP peaks is depicted as the size of the bar; RBPs shown in Figure 2G and Figure S1D are in bold. Peaks are shuffled across 3' UTRs containing eCLIP and GCLiPP peaks. Inset depicts a comparison of the scores of RBPs defined as cytosolic versus non-cytosolic as in Figure 2.

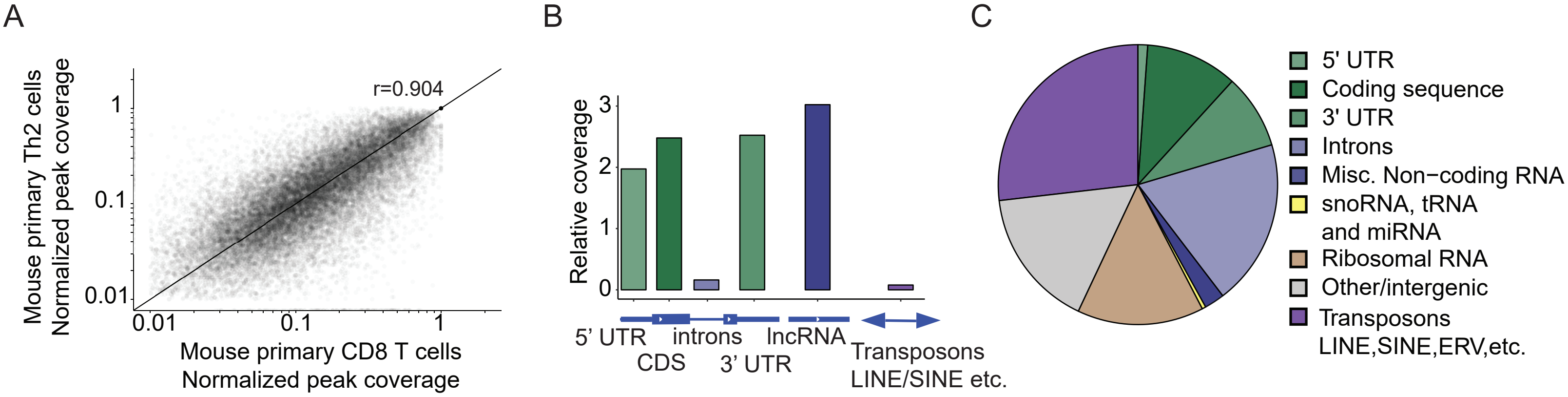

Fig S3. GCLiPP read coverage in primary mouse T cells.
(A) Normalized GCLiPP read depth (fraction of reads in called peak relative to all GCLiPP reads in annotated 3'UTR) in mouse primary Th2 and CD8 T cells. r represents Pearson correlation. (B) Proportion of mapped GCLiPP reads derived from genomic features and (C) coverage of features relative to total length of genomic features of indicated class.

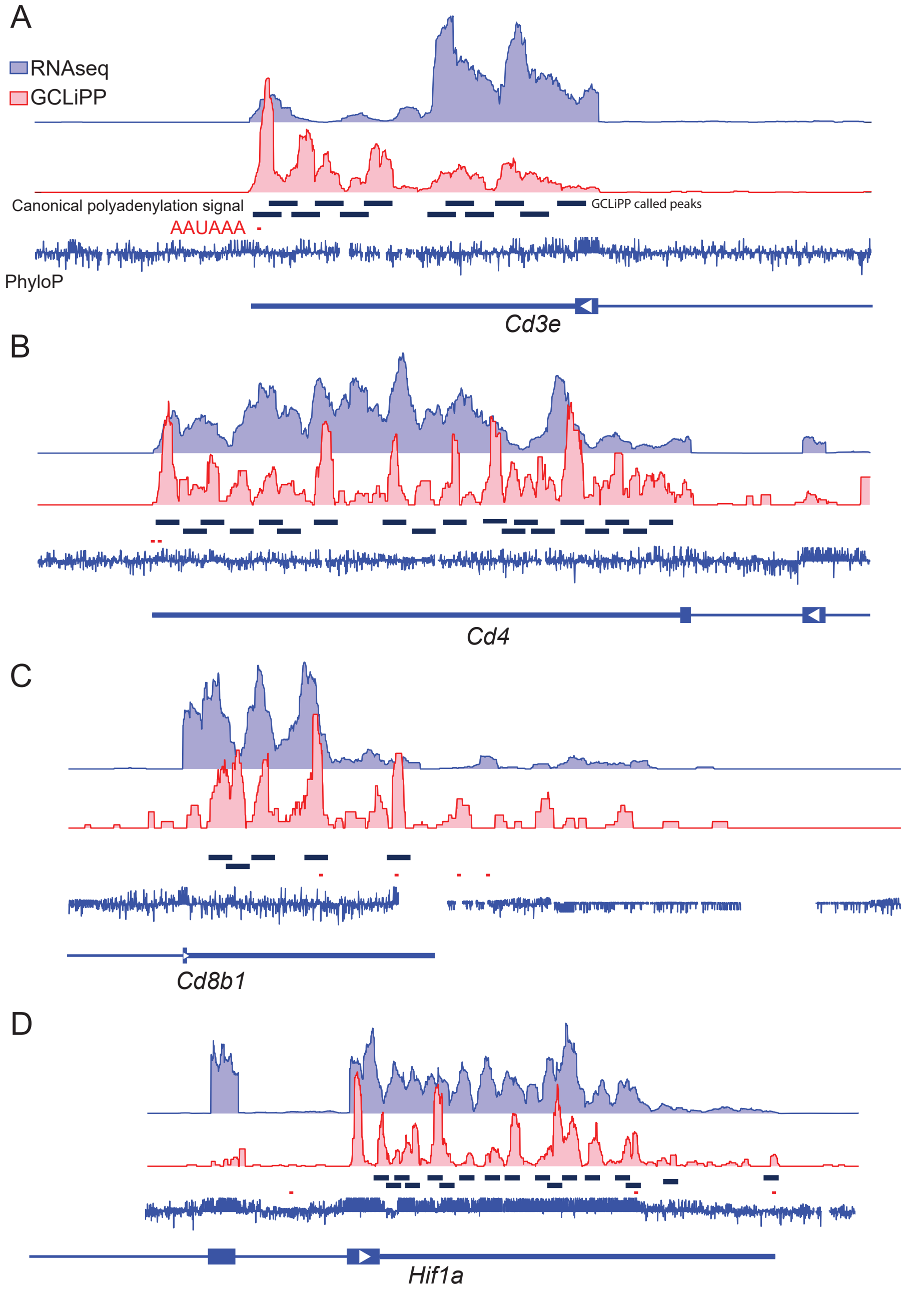

Fig S4. GCLiPP detects RBP binding of canonical polyadenylation signal. (A-D) RNAseq and GCLiPP read densities, conservation, called GCLiPP peaks and location of canonical polyadenylation signals (red lines) for indicated genes.

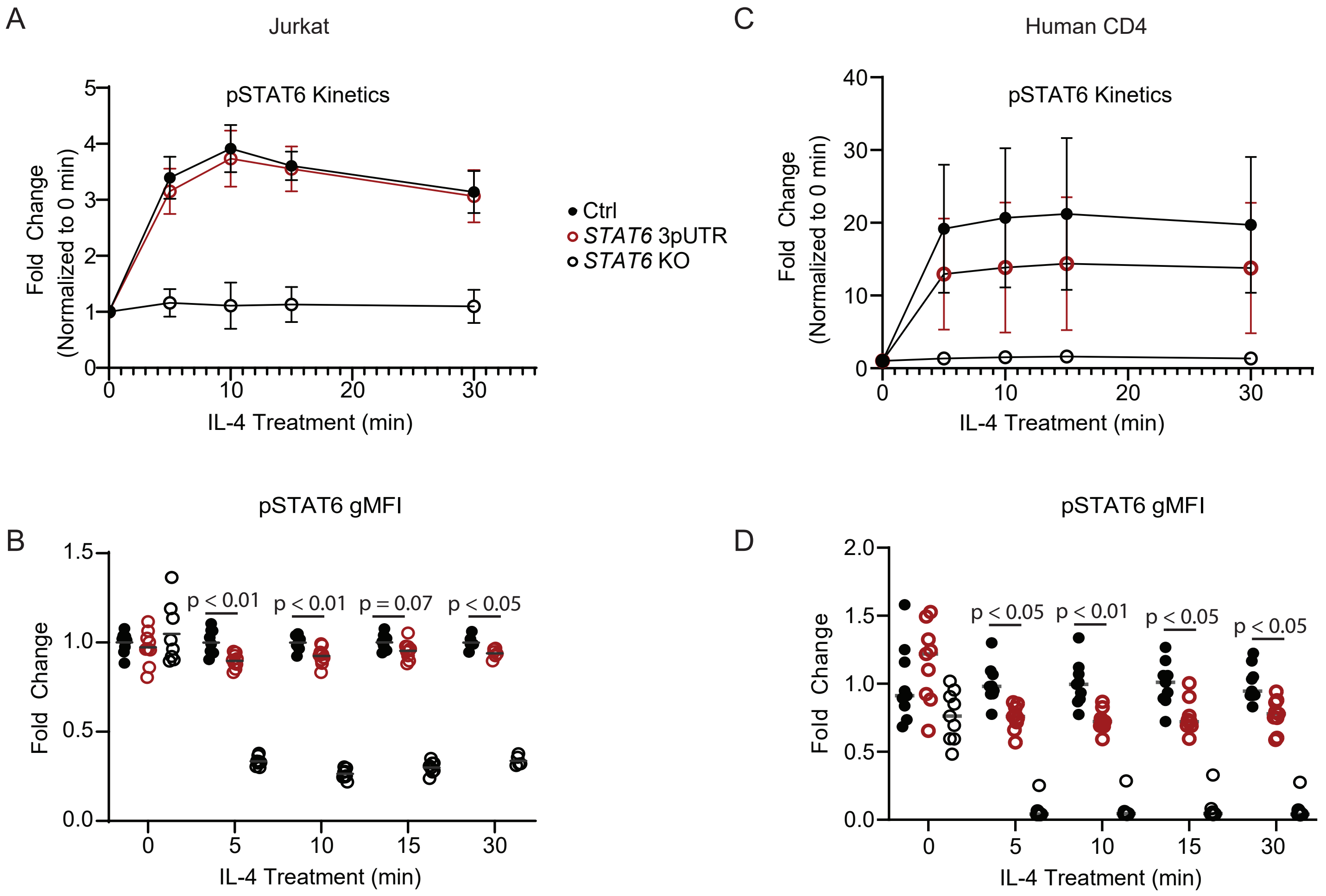

Fig S5. STAT6 expression in 3'UTR edited Jurkat cells and primary and primary human CD4 T cells.
(A) Change in pSTAT6 gMFI at 0, 5, 10, 15 and 30min relative to 0min for non-targeting control, STAT6 3'UTR-edited and STAT6 knockout (KO) Jurkat cells. **(B)** Change in pSTAT6 gMFI normalized to control within individual timepoints for all Jurkat groups. **(C-D)** Change in pSTAT6 expression in primary human CD4 T cells polarized to Th2 cells as performed for Jurkats in **A-B**.
