# Supplementary material for: GCLiPP: global crosslinking and protein purification method for constructing high-resolution occupancy maps for RNA binding proteins
Source: Genome Biol. 2023 Dec 7;24:281. doi: 10.1186/s13059-023-03125-2 (PMC10701951; doi:10.1186/s13059-023-03125-2)
Supplement: Supplementary file 8 — Additional file 8. Review history. [file 13059_2023_3125_MOESM8_ESM.docx]

**1^st^ round**

**Reviewer 1**

In this work, Zhu et al. employ an optimized technique, GCLiPP, to detect the occupancy of RBPs on poly-adenylated RNAs in two T cell models - human Jurkat T cells and mouse primary T cells. T cell activation relies heavily on gene regulation at the post-transcriptional level, making these cells especially relevant models for mapping RNA cis regulatory sequences. They found that many homologous 3' UTR regions shared GCLiPP signals between human and mouse cells, of which many preferentially occur in genes that encode proteins involved in post-transcriptional gene regulation. Subsequently, they targeted genes encoding immune-relevant transcripts by CRISPR/Cas9 to assess the functional importance of specific 3' UTR sites mapped by GCLiPP.

Overall, the manuscript is clear, well-conceptualized and well-written. It contains useful data on potential cis-regulatory RNA sequences in T cells of different species and provides insights on their evolutionary conservation. The authors provide examples of testable hypothesis generated based on the dataset and test them by editing genes of interest with the CRISPR/Cas9 system. Finally, they enable examination of their data through a web-based visualization tool (http://thagomizer.ucsf.edu), making it easy for others to access it and formulate their own testable hypotheses. However, some potential limitations of the technique need to be discussed before publication of this manuscript:

*Major comments

- The second paragraph of the introduction provides a nice review of unbiased methods for mapping protein-RNA interactions. It could be useful to preface it by first shortly introducing ''biased'', protein-centric and RNA-centric, methods (which are still used more often that the unbiased ones and could be more familiar to most people) and contrast them with the unbiased ones. It would also seem important to contrast this methodology with previously developed approaches to assess protein occupancy profiles across the transcriptome: e.g. Shueler et al. 2014 (PMID: 24417896); Ji et al. 2016 (PMID: 26900662), Huang et al. 2018 (PMID: 29636419) and Baltz et al. 2012 (already cited by the authors). Comparisons to these methods should also be commented on in the discussion.

- Poly-adenylated RNAs are selected by oligo-dT pulldown, resulting in expected enrichment of reads from mature mRNAs and long non-coding RNAs. However, ribosomal RNA also makes up a large fraction of the reads (Figure 1D, Supplementary Figure 3C). Is it expected and can this influence the overall efficiency of GCLiPP?

- Most GCLiPP signals map to single-stranded RNA regions. The authors discuss that that makes sense in the context that RBPs with ssRNA-binding RRM domains are highest expressed RBP family in Jurkat cells and that Hoefig et al., 2021 RNA interactome in T cells also predominantly detected RRM-containing RBPs. However, both that work, and this one relied on UV as the protein-RNA crosslinking method. Is it possible that this effect is partially due to a technical limitation, if UV crosslinking is more efficient for ssRNA compared to dsRNA regions?

*Minor comments

- The paper Hoefig et al., 2021 is cited as HoeFig et al., 2021 every2012where (probably a formatting mistake)

- When the authors discuss the Baltz et al 2012 paper in the introduction, they mention that it offered a global analysis of RBP binding sites, which is not really the case. This paper and that of Castello et al 2012 (also cited by the authors) were the first to identify the mRNA bound proteomes of human cells, they were not focused on identifying RBP binding sites per se. The text could be reworked to make it more precise.

**Reviewer 2**

Zhu et al provide a map of RNA binding protein occupancy of the mouse T cell transcriptome and the Jurkat T cell line. The initial part of this work focuses on the optimization and validation of the biochemical technique (GCLiPP) required to perform the mapping. While I cannot evaluate the bioinformatic effort behind it, I commend the authors for tackling an important question in the field (the complexity of RBPs decorating the 3'UTRs of transcripts in different cell types and conditions), and for introducing the important step of poly-A purification, thereby focusing only on cytoplasmic mature mRNAs, limiting the confounding aspects of RBPs involved in the nuclear steps of mRNA maturation. Their mapping results are displayed in a user-friendly online visualization tool (although, only as feedback to the authors, the tool worked well for me mostly with the RefSeq number, less well when using the gene ID). Following optimization and validation, the authors extended their analyses by comparing their data to different datasets and by taking into account conservation across species. Finally, they showed that their analyses can lead to the identification of biologically relevant processes (transcript stability/ protein expression) by RBP sites.

Please note that I cannot fully evaluate the many bioinformatics aspects of this work, and I will therefore limit my analysis to the biology and its relevance.

1) While I understand the need to use cell lines (Jurkat) in the optimization phase of a new protocol, I am less enthusiastic about the use of Jurkat data in combination with GWAS SNPs for disease susceptibility (Fig. 7). I suggest that the author add a paragraph to their discussion to outline the limitations in the interpretation of their results. This would probably also be an opportunity to describe the specific advantages and disadvantages of the GCLiPP method.

2) It is unclear why the authors decided to compare activated CD8+ T cells specifically to Th2 cells (Figure 4). Considering that these are very different types of T cells, capable of very different effector functions, it is also difficult to understand why the authors only focused on their similarities with Jurkat cells. Was anything related to the biology of these cells actually different? The legend for Figure 4 states that the tracks show the sum of Th2 and CD8 T cells together. A paragraph describing similarieties/ differences between CD8+ and Th2 cells would be recommended.

**Reviewer 3**

The manuscript by Zhu, Litterman et al. describes GCLiPP, an approach to globally profile protein-bound RNA locations throughout the transcriptome. The authors show that GCLiPP appears to characterize functionally important sites that correlate with sites of RNA binding protein association, and then show that GCLiPP can be used to explore differences between activated and resting T cells and between human and mouse. Overall I think the technique is quiet interesting and well-described, and seems to do a solid job of capturing interesting regulatory sites at a transcriptome-wide scale.
I think my main big-picture issue with the manuscript is that after reading, I'm still not very clear on what the difference is between GCLiPP and previous methods (XRNAX/OOPS) that also globally profile RNP complexes; I agree with the authors' note in the intro that those methods may have primarily focused on identifying RNA-bound proteins, but I do think that those would represent the current state-of-the-art methods for doing this, and my challenge here is that I still don't have a good sense of whether the authors are presenting GCLiPP as just another way to achieve the same data, or whether they believe that GCLiPP is better than or resolves some technical challenges or limitations with those approaches. The results here seem to be more specific & more clearly tied to RBP association sites than many of those datasets, so it's possible that GCLiPP is actually better at resolving these sites; but I don't think this is clearly stated (or shown)
Other comments:
I'd be curious to see the distribution of peaks for Fig 1D rather than reads; because the read distribution to me seems actually surprisingly poorly enriched (or perhaps more specifically, has a much higher fraction of rRNA & intron) than I'd expect for an oligo-dT pulldown RNA-seq.
I'm also unclear whether there is a paired RNA-seq control that's getting incorporated into the peak calling & analysis? What does the distribution in 1D look like for an RNA-seq library prepared using the same library preparation strategy (oligo dT selection -> T1 fragmentation -> linker ligation & library prep)? I think having some idea of this is valuable, because my experience is that any RNA-seq preparation method will have 'peaks' simply due to rnase fragmentation / ligation / mappability / etc biases, and I'm unclear how those are being controlled for in the current analysis.
As part of this, I'd also recommend a much clearer re-write of the peak calling section of the methods and more intermediate figures / datapoints, because I'm struggling to understand exactly how GCLiPP peaks were called - if I'm reading it correctly it seems like it relies on the assumption that within a 70nt window, read density will fall off as a normal distribution, but is this empirically true? (I have doubts given that a single read of 20nt will extend ~1/3 of this length, so I'd expect the distribution to have a wider peak than a normal distribution? But beyond that, I wonder if this peak-calling approach specifically selects for things with a 'peak'-like shape, and artificially avoids coated regions bound by multiple neighboring RBPs (since they won't have the valleys that seem to be necessary to call a peak using this approach)). Similarly, the authors note using effectively a 2nt UMI to identify PCR duplicates - how well does this work / what is the PCR duplication rate they observe?
Fig 2A - I think some background is desired here, because although the authors refer to this figure as 'an enrichment for ssRNA at the center of GCLiPP peaks', I'd almost argue the opposite - it seems to suggest that GCLiPP peaks are basically double-stranded regions with a small ssRNA loop at the center? The ssRNA part is almost the trivial part there (since UV only crosslinks to ssRNA, so the peak center should trivially be ssRNA-heavy), I'm more surprised by the base-pairing probability spike around that central region; but this may be a background issue, as I don't have a good sense of whether that 0.005 probability is above or below the background level for any 3'UTR region.
Fig 2B-C seem a bit weird to me; if this is going to be included it seems to make more sense to do this from quantitative mass spec data (I'd be surprised if that doesn't exist for this sample type at this point), as that would more directly reflect what the authors seem to be implying than either # of proteins or RNA quantity

I'd rename (and be a bit more careful about discussing) the results from the 'cytoplasmic RBPs' in Fig. 2H / Sup 1E - I appreciate that the authors used an unbiased source to define the grouping, but Fig 2H 'cytoplasmic RBPs' suggests that they are predominantly cytoplasmic but includes a number of RBPs that I'd argue are canonically nuclear (e.g. NCBP2 (nuclear cap binding protein 2), RBFOX2, EFTUD2, DHX30, LARP7)… maybe something like 'RBPs present in cytoplasm'?
Though as perhaps a broader question - I'd intuitively expect that the eCLIP inputs would correlate best with GCLiPP signal, as those are seemingly profiling the same thing (RBP-crosslinked RNA, with no real selection for individual RBPs)… what does the correlation look like between just the inputs and GCLiPP? The individual examples shown looks surprisingly poor, and I'm not actually sure how to interpret that
I think some alternative visualizations in Fig 6D-G would be helpful; there's a lot of data here to essentially conclude 'largely concordant patterns' - but that's kind of superficial and I don't have a good intuitive sense for what degree of similarity or difference would lead to a different conclusion. Th analysis also focuses on ARE 1 vs 2 (panels L/M), but I'd almost find it more interesting to compare against 'other deletions' - since panel F seems to suggest that ARE1 may have an opposite (stabilizing) function (at least in human)? (Also to clarify labels - Panel F is 'combined' of what? Both replicates of E? or both differential expression and GGLiPP (panels E and D))?
Figures with GCLiPP data (especially notable Fig. 7 B/D/F) should really include the location of the GCLiPP peaks identified, since they're mentioned in the text but not actually then shown in the figures as far as I can tell


Minor comments
"we systematically compared GCLiPP occupancy maps and eCLIP analyses of RBP binding profiles from ENCORE (Sundararaman et al., 2016)." - that's the wrong ref (repeated incorrectly in the discussion), should probably be the Van Nostrand et al (2020) paper also cited in the manuscript
I'd weaken the conclusion for Fig 3 to something like "Together, these data indicate that global RBP occupancy in Jurkat T cells may be partly altered by activation-induced changes in RBP expression and PAS selection." - I don't think this is a major enough issue to require additional experiments / analysis for the scope of this manuscript, but I think additional ones would be required to actually prove that these factors are actually driving the changes (particularly as the authors note that PABPC proteins don't show altered expression), particularly showing that the altered PAS sites themselves actually are the ones that contain PABPC motifs & GCLiPP / eCLIP binding sites
Are there particular transposable elements enriched in the mouse T cell experiments (Sup. Fig. 3C)? Although the authors note the potential of an annotation difference, my experience is actually the opposite (that the human annotations of transposable / repetitive elements tend to be better and more extensive than mouse), so I wonder if there's some other reason driving this (or potentially real biology, as primary T cells may actually express more of these elements in cytoplasmic mRNAs).
"A previously developed algorithm, Probabilistic Identification of Casual SNPs (PICS2) (Farh et al., 2015)" - I think that is a typo (should be PICS)

**Authors’ response**

Reviewer 1

**1. The second paragraph of the introduction provides a nice review of unbiased methods for mapping protein-RNA interactions. It could be useful to preface it by first shortly introducing ''biased'', protein-centric and RNA-centric, methods (which are still used more often that the unbiased ones and could be more familiar to most people) and contrast them with the unbiased ones. It would also seem important to contrast this methodology with previously developed approaches to assess protein occupancy profiles across the transcriptome: e.g. Shueler et al. 2014 (PMID: 24417896); Ji et al. 2016 (PMID: 26900662), Huang et al. 2018 (PMID: 29636419) and Baltz et al. 2012 (already cited by the authors). Comparisons to these methods should also be commented on in the discussion.**

Thank you for the thoughtful suggestion. We added a comparison of the methods to the discussion and included 2 new citations in the manuscript (Shueler et al and Huang et al).

*“There are certain limitations to the RBP binding sites that are captured by GCLiPP including preferential capture of polyadenylated transcripts. This contrasts against methods utilizing proximity-based CLIP [Huang et al. 2018], locked nucleic acid (LNA) capture probes [Perrez Perri et al. 2018] or organic phase separation [Queiroz et al. 2019; Trendel et al. 2019] which can capture diverse groups of non-polyadenylated noncoding RNAs. Future iterations of GCLiPP can include LNA probes to diversify the types of transcripts captured, as well as a ribosomal depletion step, to limit rRNA in the sample and enrich for other mature or noncoding transcripts. Similar to other RNA-IC and phase separation techniques, GCLiPP relies on UV cross-linking to isolate RBPs which likely captures ssRNA and under-samples other double stranded cis-regulatory regions. Additional studies utilizing modified GCLiPP protocol as well as other unbiased approaches on primary T cells will help with broadening and refining our understanding of the transcriptomic RBP binding profile.”*

**2. Poly-adenylated RNAs are selected by oligo-dT pulldown, resulting in expected enrichment of reads from mature mRNAs and long non-coding RNAs. However, ribosomal RNA also makes up a large fraction of the reads (Figure 1D, Supplementary Figure 3C). Is it expected and can this influence the overall efficiency of GCLiPP?**

Despite enrichment with oligo-dT pulldown, we expect a certain amount of rRNA reads in our  method, which does not exclude ribosomal proteins. As the reviewer states, this can influence  the expected reads and read depth of other genomic features including 3’UTRs. Future studies  can include a ribosomal depletion step to enrich for RBPs other than the ribosomal proteins. A  sentence was added in the discussion to include this point.

“*Future iterations of GCLiPP can include LNA probes to diversify the types of transcripts captured, as well as a ribosomal depletion step, to limit rRNA in the sample and enrich for other mature or noncoding transcripts.”*

**3. Most GCLiPP signals map to single-stranded RNA regions. The authors discuss that that makes sense in the context that RBPs with ssRNA-binding RRM domains are highest expressed RBP family in Jurkat cells and that Hoefig et al., 2021 RNA interactome in T cells also predominantly detected RRM-containing RBPs. However, both that work, and this one relied on UV as the protein-RNA crosslinking method. Is it possible that this**

**effect is partially due to a technical limitation, if UV crosslinking is more efficient for ssRNA compared to dsRNA regions?**

As the reviewer suggested, the predominance of ssRNA in GCLiPP peaks may be partially due  to UV crosslinking. We reference this limitation in the discussion:

*“Similar to other RNA-IC and phase separation techniques, GCLiPP relies on UV cross-linking to isolate RBPs which likely captures ssRNA and under-samples other double-stranded cis regulatory regions. Additional studies utilizing modified GCLiPP protocol as well as other unbiased approaches on primary T cells will help with broadening and refining our understanding of the transcriptomic RBP binding profile.”*

And also provide context for our findings in the results section:

*“UV crosslinking bias may drive ssRNA capture, however this enrichment in GCLiPP peaks was  consistent with high expression of RBPs with ssRNA-binding RNA recognition motif (RRM)  domains in Jurkat cells (Fig. 2B). Proteomics data from similar RNA interactome capture (RNA IC) method in primary human CD4 T cells (HoeFig. et al., 2021) also captured RBPs that  predominantly contained the RRM motif compared to other domains (Fig. 2C).”*

***Minor comments**

**The paper Hoefig et al., 2021 is cited as HoeFig et al., 2021 everywhere (probably a formatting mistake)**

We thank the reviewer for pointing out this error and have fixed it in the text.

**When the authors discuss the Baltz et al 2012 paper in the introduction, they mention that it offered a global analysis of RBP binding sites, which is not really the case. This paper and that of Castello et al 2012 (also cited by the authors) were the first to identify the mRNA bound proteomes of human cells, they were not focused on identifying RBP binding sites per se. The text could be reworked to make it more precise.**

In Baltz et al. 2012 paper, figures 6 and 7 describe the authors’ approach and analysis of global RBP binding using 4SU labeling and oligo-dT purification to capture protein-bound transcripts. Although it was not the main focus of the paper, we cite it to highlight previous work analyzing global protein binding in human cell lines. We have moved the Castello et al. 2012 citation to paragraph 3 to better reflect its contribution towards expanding the mRNA proteome as the reviewer suggested.

Reviewer 2

**1) While I understand the need to use cell lines (Jurkat) in the optimization phase of a new protocol, I am less enthusiastic about the use of Jurkat data in combination with GWAS SNPs for disease susceptibility (Fig. 7). I suggest that the author add a paragraph to their discussion to outline the limitations in the interpretation of their results. This would probably also be an opportunity to describe the specific advantages and disadvantages of the GCLiPP method.**

We thank the reviewer for highlighting the importance of confirming our results in primary  human T cells. We have included new data in Fig 7 that shows similar effects of RNA cis-

regulatory element deletion on CD5 and pSTAT6 expression in both Jurkats and primary human  CD4 T cells. These new data are described in the last subsection of the Results.

These data, together with prior work, demonstrate that Jurkats are a useful model for studying  regulatory mechanisms governing RNA binding proteins in T cells. Post-transcriptional splicing  of CD45RA to CD45RO is a good prior example of using Jurkats as model to study a T cell  function that is shared with primary human T cells (Oberdoerffer et al, Science 321:686-91; Cole  et al, RNA 21:2053-66, 2015). Our analysis of GWAS SNPs and Jurkat RBP binding profile  specifically investigates genes that are expressed in the cell line and primary T cells to exclude  cell-specific bias. We added a sentence to acknowledge this in the discussion:

*“Our data uncovered conserved regulatory activity in the dissected 3’UTRs in both Jurkats and  primary human T cells, demonstrating the utility of using Jurkat RBP binding data to guide  discovery of post-transcriptional elements for shared expressed genes in primary T cells.”*

**2) It is unclear why the authors decided to compare activated CD8+ T cells specifically to Th2 cells (Figure 4). Considering that these are very different types of T cells, capable of very different effector functions, it is also difficult to understand why the authors only focused on their similarities with Jurkat cells. Was anything related to the biology of these cells actually different? The legend for Figure 4 states that the tracks show the sum of Th2 and CD8 T cells together. A paragraph describing similarieties/ differences between CD8+ and Th2 cells would be recommended.**

We agree with the reviewer that further work is warranted to discover cell-type specific RBP occupancy profiles. We hope to expand this work in that direction in the future, based on the data presented in this manuscript. Our preliminary analysis of these two highly related cell types (CD8 and Th2) yielded few cell-type specific differences. Most differences observed could be attributed to differentially expressed transcripts rather than differences in RBP occupancy on those transcripts. A sentence was added in results section briefly explaining the function of CD8 and Th2 cells. Future studies should examine cell type differences in more diverse cell types.

*“These two subsets of T cells perform different functions with CD8 T cells involved in cell mediated immunity and Th2 involved in orchestrating barrier immunity. Despite these differences, the cells share core T cell machinery and were treated as a broader group of primary mouse T cells for following analyses.”*

We focused on similarities between Jurkat T cells and mouse primary T cells because of our interest in evolutionarily conserved RNA circuits (e.g. the regulatory elements in Pim3, and motifs within conserved peaks). The primary cell data could undoubtedly provide additional insights not adequately modeled in Jurkats. In this work, we leveraged the advantage of the human origin of Jurkat cells to prioritize RBP-occupied regions implicated in human disease.

Reviewer 3

**I think my main big-picture issue with the manuscript is that after reading, I'm still not very clear on what the difference is between GCLiPP and previous methods (XRNAX/OOPS) that also globally profile RNP complexes; I agree with the authors' note in the intro that those methods may have primarily focused on identifying RNA-bound proteins, but I do think that those would represent the current state-of-the-art methods for doing this, and my challenge here is that I still don't have a good sense of whether the authors are presenting GCLiPP as just another way to achieve the same data, or whether they believe that GCLiPP is better than or resolves some technical challenges or limitations with those approaches. The results here seem to be more specific & more**

**clearly tied to RBP association sites than many of those datasets, so it's possible that GCLiPP is actually better at resolving these sites; but I don't think this is clearly stated (or shown)**

We have modified the manuscript to reflect its submission as a Research article. GCLiPP was used to generate a RBP binding profile of the T cell transcriptome to investigate interesting T cell biology rather than optimized to overcome limitations of previous methods such as XRNAX and OOPS. Our data indicate that GCLiPP has advantages over some of these methods (e.g. high resolution as indicated by conservation of detected binding peaks, selection for polyadenylated transcripts (which could be an advantage or disadvantage, depending on the research question)). However, we do not claim some kind objective superiority for our technique. We have added a comparison of our method against others in the discussion:

*“There are certain limitations to the RBP binding sites that are captured by GCLiPP including preferential capture of polyadenylated transcripts. This contrasts against methods utilizing proximity-based CLIP [Huang et al. 2018], locked nucleic acid (LNA) capture probes [Perrez Perri et al. 2018] or organic phase separation [Queiroz et al. 2019; Trendel et al. 2019] which can capture diverse groups of non-polyadenylated noncoding RNAs. Future iterations of GCLiPP can include LNA probes to diversify the types of transcripts captured, as well as a ribosomal depletion step, to limit rRNA in the sample and enrich for other mature or noncoding transcripts. Similar to other RNA-IC and phase separation techniques, GCLiPP relies on UV cross-linking to isolate RBPs which likely captures ssRNA and under-samples other double stranded cis-regulatory regions. Additional studies utilizing modified GCLiPP protocol as well as other unbiased approaches on primary T cells will help with broadening and refining our understanding of the transcriptomic RBP binding profile.”*

Other comments:

**I'd be curious to see the distribution of peaks for Fig 1D rather than reads; because the read distribution to me seems actually surprisingly poorly enriched (or perhaps more specifically, has a much higher fraction of rRNA & intron) than I'd expect for an oligo-dT pulldown RNA-seq.**

We called peaks only in 3’ UTRs. The abundance and repetitive nature of rRNA loci preclude  meaningful analysis of GCLiPP peaks. Introns occupy a larger genomic space and were  sparsely decorated with GCLiPP reads (e.g. see Fig. 4B-F). We added a discussion point on the  limitations of GCLiPP, and modifications that could be made to enrich for mature transcripts.

*“Future iterations of GCLiPP can include LNA probes to diversify the types of transcripts captured, as well as a ribosomal depletion step, to limit rRNA in the sample and enrich for other mature or noncoding transcripts.”*

**I'm also unclear whether there is a paired RNA-seq control that's getting incorporated into the peak calling & analysis? What does the distribution in 1D look like for an RNA seq library prepared using the same library preparation strategy (oligo dT selection -> T1 fragmentation -> linker ligation & library prep)? I think having some idea of this is valuable, because my experience is that any RNA-seq preparation method will have 'peaks' simply due to rnase fragmentation / ligation / mappability / etc biases, and I'm unclear how those are being controlled for in the current analysis.**

We did perform RNA-seq in parallel for the primary mouse T cell samples. As the reviewer notes, RNA-seq also generates non-random read density with apparent peaks which likely reflect biases in fragmentation and library generation. These patterns did not resemble the

corresponding GCLiPP data (e.g. see Fig. 4B-F). However, RNA-seq libraries are of course prepared with a very different protocol. We attempted to generate a “background” data set by generating a library from RNAseT1-digested RNA from Jurkat cells that were not irradiated to crosslink protein and RNA. However, this protocol yields very little RNA, as one would expect (Fig. 1B). We did consider the biases that could be present in this technique, including the preference for single-stranded RNA capture in UV crosslinking, and added a discussion point to acknowledge this limitation.

*“Similar to other RNA-IC and phase separation techniques, GCLiPP relies on UV cross-linking to isolate RBPs which likely captures ssRNA and under-samples other double-stranded cis regulatory regions. Additional studies utilizing modified GCLiPP protocol as well as other unbiased approaches on primary T cells will help with broadening and refining our understanding of the transcriptomic RBP binding profile.”*

**As part of this, I'd also recommend a much clearer re-write of the peak calling section of the methods and more intermediate figures / datapoints, because I'm struggling to understand exactly how GCLiPP peaks were called - if I'm reading it correctly it seems like it relies on the assumption that within a 70nt window, read density will fall off as a normal distribution, but is this empirically true? (I have doubts given that a single read of 20nt will extend ~1/3 of this length, so I'd expect the distribution to have a wider peak than a normal distribution? But beyond that, I wonder if this peak-calling approach specifically selects for things with a 'peak'-like shape, and artificially avoids coated regions bound by multiple neighboring RBPs (since they won't have the valleys that seem to be necessary to call a peak using this approach)). Similarly, the authors note using effectively a 2nt UMI to identify PCR duplicates - how well does this work / what is the PCR duplication rate they observe?**

We thank the reviewer for raising this important issue. The peak-calling method used is as simple as the reviewer suggests – we modeled a normal distribution across different lengths (window sizes) of RNA. As the reviewer suggests, this biases detection to peak-like shapes and may miss other RBP binding regions that may present as other patterns. The chosen 70nt window reflects the resolution of the assay based on the distribution of reads across the transcriptome, reinforced by the analysis of phylogenetic conservation (Fig. 2D). Importantly, this analysis is not circular, as we utilized a distinct algorithm, CLIPper, which defines peak regions (“clusters”) by fitting the genomic read profiles to splines. This approach has advantages and disadvantages as well. Future work is needed to further optimize peak calling in CLIP and GCLiPP data. For comparative analyses, we have employed methods that do not require peak calling (e.g. Fig. 3).

We agree that a longer UMI would improve GCLiPP. Our unjackpotting script does remove many duplicates that are present in the dataset and eliminates artefactual blocks of duplicate reads. With deeper sequencing of the GCLiPP libraries, a more complex UMI will be important.

**Fig 2A - I think some background is desired here, because although the authors refer to this figure as 'an enrichment for ssRNA at the center of GCLiPP peaks', I'd almost argue the opposite - it seems to suggest that GCLiPP peaks are basically double-stranded regions with a small ssRNA loop at the center? The ssRNA part is almost the trivial part there (since UV only crosslinks to ssRNA, so the peak center should trivially be ssRNA heavy), I'm more surprised by the base-pairing probability spike around that central region; but this may be a background issue, as I don't have a good sense of whether that 0.005 probability is above or below the background level for any 3'UTR region.**

3’ UTRs are enriched for U rich sequences, and therefore have a low overall propensity for  folding into dsRNA structures. In prior work, we and others showed that the destabilizing activity  of dsRNA may underlie this evolutionary outcome (Litterman et al, Genome Research 2019;  Rabani et al, Molecular Cell 2017). The 0.005 probability of folding is the background level for 3’  UTRs. The heat map in Fig. 2A was sized to include enough surrounding sequence to extend  beyond the GCLiPP peaks to indicate this background level. Extending to 200 nt simply  extended this band of ~0.005 probability of folding.

**Fig 2B-C seem a bit weird to me; if this is going to be included it seems to make more sense to do this from quantitative mass spec data (I'd be surprised if that doesn't exist for this sample type at this point), as that would more directly reflect what the authors seem to be implying than either # of proteins or RNA quantity**

We thank the reviewer for their advice. The human RNA-IC proteomics study used peptide  quantity data to compare non-crosslinked to crosslinked samples to determine which proteins  are RNA-bound in primary human T cells. We mirrored this analysis to quantify the number of RBPs representing different families. Assuming that RNA quantity at least broadly correlates  with protein abundance, the Jurkat RNA-seq data further support the preponderance of RRM  proteins among RBPs expressed in T cells.

**I'd rename (and be a bit more careful about discussing) the results from the 'cytoplasmic RBPs' in Fig. 2H / Sup 1E - I appreciate that the authors used an unbiased source to define the grouping, but Fig 2H 'cytoplasmic RBPs' suggests that they are predominantly cytoplasmic but includes a number of RBPs that I'd argue are canonically nuclear (e.g. NCBP2 (nuclear cap binding protein 2), RBFOX2, EFTUD2, DHX30, LARP7)… maybe something like 'RBPs present in cytoplasm'?**

We have changed the title of Fig 2H to reflect the reviewer’s advice; indeed, many RBPs shuttle  between cytoplasm and nucleus, and those selected in this analysis were flagged as  predominantly cytoplasmic in the COMPARTMENTS database.

**Though as perhaps a broader question - I'd intuitively expect that the eCLIP inputs would correlate best with GCLiPP signal, as those are seemingly profiling the same thing (RBP crosslinked RNA, with no real selection for individual RBPs)… what does the correlation**

**look like between just the inputs and GCLiPP? The individual examples shown looks surprisingly poor, and I'm not actually sure how to interpret that**

Comparison of input and GCLiPP is depicted in Fig 2D and Fig S1C and exhibited weak  correlation. The eCLIP input is RNA isolated using IgG antibody control and acts as a  background dataset for random RNAs that bind non-specifically during the eCLIP procedure.  We therefore do not expect GCLiPP, which can pull out real RBP-RNA interactions, to be similar  to a background sample.

**I think some alternative visualizations in Fig 6D-G would be helpful; there's a lot of data here to essentially conclude 'largely concordant patterns' - but that's kind of superficial and I don't have a good intuitive sense for what degree of similarity or difference would lead to a different conclusion. Th analysis also focuses on ARE 1 vs 2 (panels L/M), but I'd almost find it more interesting to compare against 'other deletions' - since panel F**

**seems to suggest that ARE1 may have an opposite (stabilizing) function (at least in human)? (Also to clarify labels - Panel F is 'combined' of what? Both replicates of E? or both differential expression and GGLiPP (panels E and D))?**

We thank the reviewer for their thoughtful insight and questions. Our CRISPR dissection data reveals destabilizing activity on a per nucleotide basis and can be used to understand the regulatory activity of ARE1 and ARE2 in comparison to each other and other regions in the 3’UTR. Indeed, ARE1 appears in a region that may have stabilizing activity relative to other sequences in the Pim3 3’UTR. Our current work focuses on how biochemical maps can provide more targeted discovery of functional elements in addition to using RBP motifs. The Y-axis label of Fig. 6F denotes combined p-value for both replicates of Fig. 6E. We clarified this in the figure legend.

**Figures with GCLiPP data (especially notable Fig. 7 B/D/F) should really include the location of the GCLiPP peaks identified, since they're mentioned in the text but not actually then shown in the figures as far as I can tell**

Figures 7B, E, and H have arrow heads that mark gRNA placement for CRISPR deletion. Due to design constraints for gRNAs, we were unable to precisely delete GCLiPP peaks. As such, we refer to these deleted areas as “regions” or “GCLiPP-identified RBP binding regions”. We edited the discussion to correct one instance where the term “peak” was used incorrectly. These regions often contain multiple peaks, and we cannot attribute with certainty the observed regulatory activity to the peak that directly overlaps the disease-associated SNP (which is marked with a vertical dashed line). As such, we prefer to emphasize these regions of RBP binding instead of focusing attention on the single peak containing the SNP.

**Minor comments**

**"we systematically compared GCLiPP occupancy maps and eCLIP analyses of RBP binding profiles from ENCORE (Sundararaman et al., 2016)." - that's the wrong ref (repeated incorrectly in the discussion), should probably be the Van Nostrand et al (2020) paper also cited in the manuscript**

The datasets used in our analysis was from Sundararamen et al. 2016 and does not include the  updated database cited in Van Nostrand et al. 2020, which is why we included the 2016 citation.

**I'd weaken the conclusion for Fig 3 to something like "Together, these data indicate that global RBP occupancy in Jurkat T cells may be partly altered by activation-induced changes in RBP expression and PAS selection." - I don't think this is a major enough issue to require additional experiments / analysis for the scope of this manuscript, but I think additional ones would be required to actually prove that these factors are actually driving the changes (particularly as the authors note that PABPC proteins don't show altered expression), particularly showing that the altered PAS sites themselves actually are the ones that contain PABPC motifs & GCLiPP / eCLIP binding sites**

We have changed the text in the results (highlighted) as the reviewer suggested.

*“Together, these data indicate that global RBP occupancy in Jurkat T cells* may *be altered by  activation-induced changes in RBP expression and PAS selection.”*

**Are there particular transposable elements enriched in the mouse T cell experiments (Sup. Fig. 3C)? Although the authors note the potential of an annotation difference, my experience is actually the opposite (that the human annotations of transposable / repetitive elements tend to be better and more extensive than mouse), so I wonder if there's some other reason driving this (or potentially real biology, as primary T cells may actually express more of these elements in cytoplasmic mRNAs).**

The reviewer suggests an interesting point. Future experiments beyond the scope of the paper can examine the transposable elements in mouse and human T cells to elucidate our observation. The new T2T genome build will aid in this analysis, though the use of small RNA libraries with short reads may limit our ability to accurately map GCLiPP signal to specific repeat sequences in the transcriptome.

**"A previously developed algorithm, Probabilistic Identification of Casual SNPs (PICS2) (Farh et al., 2015)" - I think that is a typo (should be PICS)**

This was corrected in the text.

**2^nd^ round**

**Reviewer 1**

I am satisfied with the improvements made by the authors.

**Reviewer 2**

In this revised version of their work, the authors added experiments using primary human T lymphocytes, showing similar results to Jurkat cells, and addressed by concerns.

**Reviewer 3**

I appreciate the author’s responses and edits to the text

I’d appreciate a bit more discussion of the causality the authors believe in Fig 7 - the paragraph starts by saying “SNP rs1059513 … making it by far the most likely causal variant in the locus for this trait.“, but in 7H-J the SNP is ~100nt away from the spike in GCLiPP signal and the deletion is a ~250nt window that includes both. Do the authors ultimately think that that SNP is causal and the site of RBP association but the GCLiPP peak is shifted for some reason? Or is the GCLiPP peak the actual element and the SNP is just linked?

To the prior comment & response <<Though as perhaps a broader question - I'd intuitively expect that the eCLIP inputs would correlate best with GCLiPP signal, as those are seemingly profiling the same thing (RBP- crosslinked RNA, with no real selection for individual RBPs)... what does the correlation look like between just the inputs and GCLiPP? The individual examples shown looks surprisingly poor, and I'm not actually sure how to interpret that

Comparison of input and GCLiPP is depicted in Fig 2D and Fig S1C and exhibited weak correlation. The eCLIP input is RNA isolated using IgG antibody control and acts as a background dataset for random RNAs that bind non-specifically during the eCLIP procedure. We therefore do not expect GCLiPP, which can pull out real RBP-RNA interactions, to be similar to a background sample. >>

This is not correct; the ENCORE eCLIP inputs are lysate that is run through denaturing SDS-PAGE, nitrocellulose membrane transfer, isolation of a size-matched region of the membrane, and subsequent RNA isolation and library preparation – so they are selecting only for protein-bound RNA (in the same kDa size range as the IP).

This is my surprise – in theory it seems like this should correlate with the protein-bound RNA regions in GCLiPP, and so the lack of correlation to me suggests either (1) a substantial portion of the GCLiPP signal is coming from large complexes that don’t run into the correct range in the eCLIP SDS-PAGE step, which would be interesting or (2) one (or both) methods have other biases in isolation or library prep that drive big differences (which also seems interesting), and I think it would be worth some discussion as to what the authors think here

**Authors’ response**

Reviewer 3

1. I appreciate the author’s responses and edits to the text. I’d appreciate a bit more discussion of the causality the authors believe in Fig 7 - the paragraph starts by saying “SNP rs1059513 … making it by far the most likely causal variant in the locus for this trait.“, but in 7H-J the SNP is ~100nt away from the spike in GCLiPP signal and the deletion is a ~250nt window that includes both. Do the authors ultimately think that that SNP is causal and the site of RBP association but the GCLiPP peak is shifted for some reason? Or is the GCLiPP peak the actual element and the SNP is just linked?

This is an interesting point, and something we are pursuing for future studies, guided by the GCLiPP data. The PICS2 algorithm, which is a statistical model for predicting causality among linked SNPs, returns a very strong prediction of causality (>98%) for rs1059513 in the association with allergy. As shown in Figure 7H, this SNP corresponds with a local maximum of GCLiPP signal, although this is not the largest GCLiPP “peak” in the STAT6 3’ UTR, or even the largest peak in the region that we were able to target with CRISPR. At this level of dissection, we are not able to ascertain whether the SNP acts by disrupting the cis-regulatory function of the small peak that precisely coincides with the SNP, by altering local protein binding and or RNA structure to disrupt the activity of the adjacent larger peak, or both. Our prior experience with other regions and our observations of GCLiPP signal at known regulatory elements indicate that all of these are very viable possibilities.

A recent publication (Sharma et al, new ref 56 in the manuscript) showed that gain of function promoter mutations that moderately increase the expression of STAT6 lead to severe allergic disease in human patients, demonstrating that changes in STAT6 expression can be pivotal in the development of allergy, supporting the idea that the STAT6 3’ UTR polymorphism likely affects the incidence of allergy by affecting mRNA stability. Indeed, changes in any 3’ UTR cis-regulatory elements that regulate STAT6 expression would likely have this effect, of course including changes that are not represented at such high frequency in human populations as rs1059513. We will explore this further in the future, with a focus on the allergy-associated SNP as well as a broader search of RBP occupied regions in the 3’UTR.

2. To the prior comment & response <<Though as perhaps a broader question - I'd intuitively expect that the eCLIP inputs would correlate best with GCLiPP signal, as those are seemingly profiling the same thing (RBP- crosslinked RNA, with no real selection for individual RBPs)... what does the correlation look like between just the inputs and GCLiPP? The individual examples shown looks surprisingly poor, and I'm not actually sure how to interpret that. Comparison of input and GCLiPP is depicted in Fig 2D and Fig S1C and exhibited weak correlation. The eCLIP input is RNA isolated using IgG antibody control and acts as a background dataset for random RNAs that bind non-specifically during the eCLIP procedure. We therefore do not expect GCLiPP, which can pull out real RBP-RNA interactions, to be similar to a background sample. This is not correct; the ENCORE eCLIP inputs are lysate that is run through denaturing SDS-PAGE, nitrocellulose membrane transfer, isolation of a size-matched region of the membrane, and subsequent RNA isolation and library preparation – so they are selecting only for protein-bound RNA (in the same kDa size range as the IP). This is my surprise – in theory it seems like this should correlate with the protein-bound RNA regions in GCLiPP, and so the lack of correlation to me suggests either (1) a substantial portion of the GCLiPP signal is coming from large complexes that don’t run into the correct range in the eCLIP SDS-PAGE step, which would be interesting or (2) one (or both) methods have other biases in isolation or library prep that drive big differences (which also seems interesting), and I think it would be worth some discussion as to what the authors think here.

We stand corrected regarding the eCLIP control samples. We thank the reviewer, as this has helped us to sharpen the interpretation of our observations in the correlation analyses. We agree that the eCLIP control could ideally produce data very similar to GCLiPP, and there is significant correlation between the two. These data are embedded in the analysis shown in Figure 2H, where the correlation t statistic is equally good for comparing GCLiPP to eCLIP control as for protein-specific eCLIP, except for abundant cytoplasmic RBPs that make large contributions to the overall GCLiPP signal. This is exemplified by Fig. 2E; there is significant similarity between eCLIP control and GCLiPP signal across the CLIC1 3’ UTR, but even better correlation between TIA1 eCLIP and GCLiPP signal because of the strong shared peak at the 3’ end. The GCLiPP procedure does involve a gel purification step, though the region extracted is large. In addition, we suspect that the optimization of RBP:mRNA capture in the GCLiPP protocol reduces noise and variability compared to the eCLIP control protocol, and may also introduce different biases in isolation.

**3^rd^ round**

**Reviewer 3**

I appreciate the authors responses and edits, which I think help to emphasize the improved value of GCLiPP over prior methods to profile RNA binding protein-associated RNA. The improved resolution of GCLiPP should provide a useful approach across a range of areas. I appreciate the authors’ desire to avoid focusing on the shortcomings of other methods, but I do think that as a methods article, it is important to have a strong sense of the benefits of GCLiPP relative to existing state of the art. As such I have some minor suggestions to clarify and strengthen those claims.

This is partly a language suggestion – I think the “GCLiPP read density represents cytosolic RBP occupancy” section uses stronger claims than are justified (or necessary for the method and insights to be publishable). In particular, I find the use of “direct” in “We assessed the performance of GCLiPP by direct comparison with eCLIP and phase separation-based methods.” a bit strange, since the only real comparison is indirect (by using PhyloP score length distributions – but it emphasizes that including a direct comparison of read density between the 3 phase-separation methods that shows a similar tighter distribution to Fig. 2D in read density profiles would actually significantly bolster the claim here.

However, I would recommend reconfiguring Fig. 2D to make it easier for the viewer to compare the distributions (perhaps combining the positive and negative values, and then zooming in on the 0-50 region?), and including some statistical validation. It’s hard to visually compare the subtle differences between the curves (with no statistics attached), and moreover the text implies that the difference between ENCORE and GCLiPP is “a high resolution that closely resembles gold- standard eCLIP data”, but the difference between GCLiPP and XRNAX is “even broader … indicating lower resolution” – I think the displayed figure is a bit superficial to serve as the only evidence supporting this fairly major pillar of GCLiPP method comparison with XRNAX/OOPS.

**Authors’ response**

Reviewer 3

I appreciate the authors responses and edits, which I think help to emphasize the improved value of GCLiPP over prior methods to profile RNA binding protein-associated RNA. The improved resolution of GCLiPP should provide a useful approach across a range of areas. I appreciate the authors’ desire to avoid focusing on the shortcomings of other methods, but I do think that as a methods article, it is important to have a strong sense of the benefits of GCLiPP relative to existing state of the art. As such I have some minor suggestions to clarify and strengthen those claims.

We thank the reviewer for suggestions that improved the manuscript, and for their keen appreciation of the methods available for RBP occupancy mapping and the relative merits and utility of GCLiPP.

This is partly a language suggestion – I think the “GCLiPP read density represents cytosolic RBP occupancy” section uses stronger claims than are justified (or necessary for the method and insights to be publishable). In particular, I find the use of “direct” in “We assessed the performance of GCLiPP by direct comparison with eCLIP and phase separation-based methods.” a bit strange, since the only real comparison is indirect (by using PhyloP score length distributions – but it emphasizes that including a direct comparison of read density between the 3 phase-separation methods that shows a similar tighter distribution to Fig. 2D in read density profiles would actually significantly bolster the claim here.
 We removed the word “direct” as suggested, and also added additional figure panels to flesh out the comparison of resolution across assays. In the new Fig. 2D, we now show the size distribution of CLIPper-called peaks for each method (using the same CLIPper settings to call peaks in each dataset), providing a much more direct comparison of the read density. Statistics for the comparison of these distributions are not particularly meaningful with such high *n,* but they are provided in the updated text (p<10^-300^ for each pairwise comparison).

However, I would recommend reconfiguring Fig. 2D to make it easier for the viewer to compare the distributions (perhaps combining the positive and negative values, and then zooming in on the 0-50 region?), and including some statistical validation. It’s hard to visually compare the subtle differences between the curves (with no statistics attached), and moreover the text implies that the difference between ENCORE and GCLiPP is “a high resolution that closely resembles gold- standard eCLIP data”, but the difference between GCLiPP and XRNAX is “even broader … indicating lower resolution” – I think the displayed figure is a bit superficial to serve as the only evidence supporting this fairly major pillar of GCLiPP method comparison with XRNAX/OOPS.

The histograms showing PhyloP scores across 200 nt regions centered on called peaks from the former Fig. 2D are now overlaid in Fig. 2E to aid comparison, and scores for a large set of randomly chosen peaks are shown for each method above the histogram. In addition, we took reviewer’s suggestion to combine the positive and negative values (equivalent to measuring the distance from peak center, regardless of direction), and then plotted the PhyloP values at matched distances to visualize a pairwise comparison of the ENCORE eCLIP data with GCLiPP, XRNAX, and OOPS in the new Fig. 2F. This transformation also facilitated statistical analysis by linear regression for each pairing. We also considered treating the patterns in Fig. 2E as distributions with potentially different variances, and significance testing using this method returned extremely low p values. However, we rejected this idea since these data are not really distributions at all but ordered series. We think that the linear regressions provide the best statistical and visual comparison between the methods. And overall, we believe that these enhancements provide a more complete comparison of GCLiPP and phase separation methods for mapping RBP occupancy on RNA.
